# Organic radical reactivity: activation and deactivation through alkyl substitution

**DOI:** 10.1039/d6cp03338e

**Published:** 2026-09-09

**Authors:** Daniela Rodrigues Silva, J. Martijn van der Schuur, F. Matthias Bickelhaupt

**Affiliations:** a Department of Chemistry and Pharmaceutical Sciences, Amsterdam Institute of Molecular and Life, Sciences (AIMMS), Vrije Universiteit Amsterdam De Boelelaan 1108 1081 HZ Amsterdam The Netherlands f.m.bickelhaupt@vu.nl https://www.theochem.nl; b Polymer Specialties, Nouryon Zutphenseweg 10 7418 AJ Deventer The Netherlands; c Institute of Molecules and Materials, Radboud University Heyendaalseweg 135 6525 AJ Nijmegen The Netherlands; d Department of Chemical Sciences, University of Johannesburg Auckland Park Johannesburg 2006 South Africa

## Abstract

We have recently shown that alkyl substituents destabilize carbon-centered radicals, contradicting the conventional view that alkyl substitution stabilizes radicals through hyperconjugation. In this work, we show quantum chemically that these thermodynamically destabilized organic radicals are kinetically activated towards radical additions at double bonds. However, this electronic effect can be counteracted by steric repulsion in the case of sterically crowded double bonds. These findings reconcile the apparent contradiction between thermodynamic and kinetic effects.

## Abstract 2

Methyl substituents destabilize organic radicals thermodynamically, but they can either enhance or attenuate their reactivity by, respectively, destabilizing the radical's unpaired electron or aggravating their steric repulsion with the alkene.

## Graphical abstract

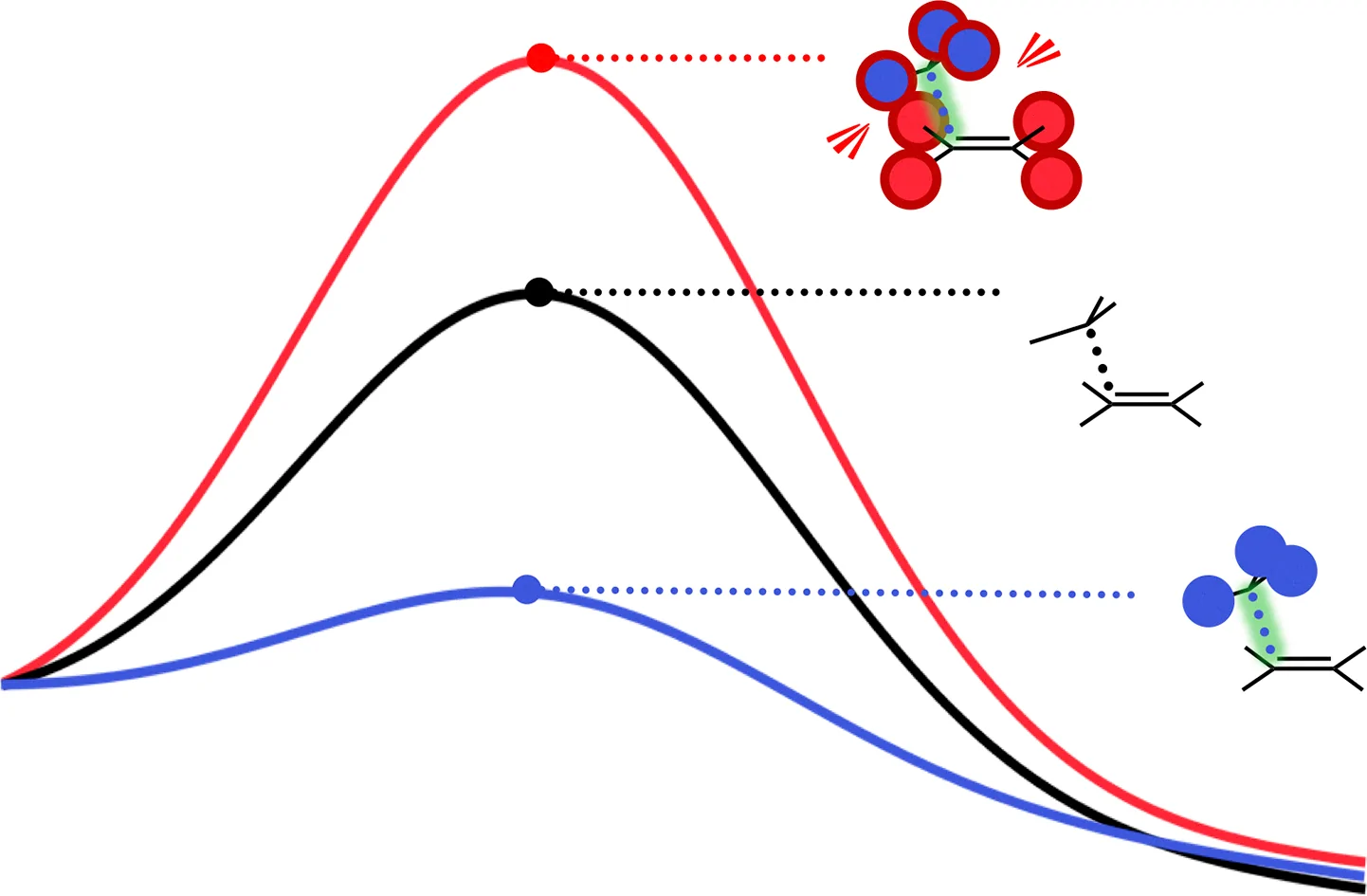

The thermodynamic stability of organic radicals, carbocations, and alkanes has been believed to increase in general with their degree of substitution, from primary to secondary to tertiary species.^1^ This was traditionally attributed to additional stabilization provided by geminal alkyl substituents through electron delocalization (*vide infra*).^2^ However, in a recent series of studies,^3^ we have shown that this picture and its rationale resulted from a misinterpretation of the metrics commonly used to quantify thermodynamic stability, such as the radical stabilization energy (RSE)^3*a*,3*b*^ and the heat of formation (Δ*H*_f_).^3*c*^ This misinterpretation arose because such metrics account for more than one effect at the same time. For example, the RSE is defined by the isodesmic reaction in eqn (1),^4^ which relates the homolytic C–H bond dissociation enthalpy of a substituted alkane (R_3_C–H) to that of methane (H_3_C–H):

1 R_3_C˙ + H_3_C–H → R_3_C–H + H_3_C˙ Δ*H* = RSE

The C–H bond in R_3_C–H becomes progressively weaker from methane (H_3_C–H) to primary (MeH_2_C–H) to secondary (Me_2_HC–H) to tertiary (Me_3_C–H) alkanes, that is, the RSE becomes more positive.^5^ This trend had been interpreted as proof that alkyl substituents stabilize radicals, as the formation of R_3_C˙ is supposedly favored over H_3_C˙. However, several authors have pointed out problems with this interpretation.^6^ Most importantly, this interpretation overlooks that the C–H bond strength trends depend on the substituent effect on both the radical R_3_C˙ and the parent molecule R_3_C–H. Contrary to the textbook rationale, alkyl substituents destabilize both species, but they destabilize the more sterically congested parent molecule R_3_C–H more than the radical R_3_C˙. This leads to the observed weakening of the C–H bond in R_3_C–H with an increasing degree of substitution.^3*a*^ In a recent paper,^3*c*^ we have provided a framework for extracting the actual substituent effect using either experimental or computational data.

Strikingly, the same electron delocalization invoked to explain the higher thermodynamic stability of alkyl-substituted radicals is also used to rationalize their enhanced reactivity (*i.e.*, kinetic instability) toward addition reactions.^7^ This electron delocalization stems from hyperconjugation interactions between the σ_C–H_ orbital of the alkyl substituents and the electron-deficient 2p_*z*_ orbital of the carbon radical center.^1^ As illustrated in Fig. 1 for the *tert*-butyl radical (Me_3_C˙), hyperconjugation stabilizes the *σ*_C–H_ electrons in the bonding molecular orbital (*i.e.*, the HOMO) of the final Me_3_C˙ radical, whereas the unpaired electron is pushed up in energy in the antibonding molecular orbital (*i.e.*, the SOMO). This is the mechanism through which electron-donating substituents, such as alkyl groups, enhance the nucleophilicity of radicals. Hence, the higher-lying SOMO engages in more stabilizing interactions with the LUMO of the alkene, the so-called polar effect, which stabilizes the transition state of radical addition reactions.^8^

**Fig. 1:**
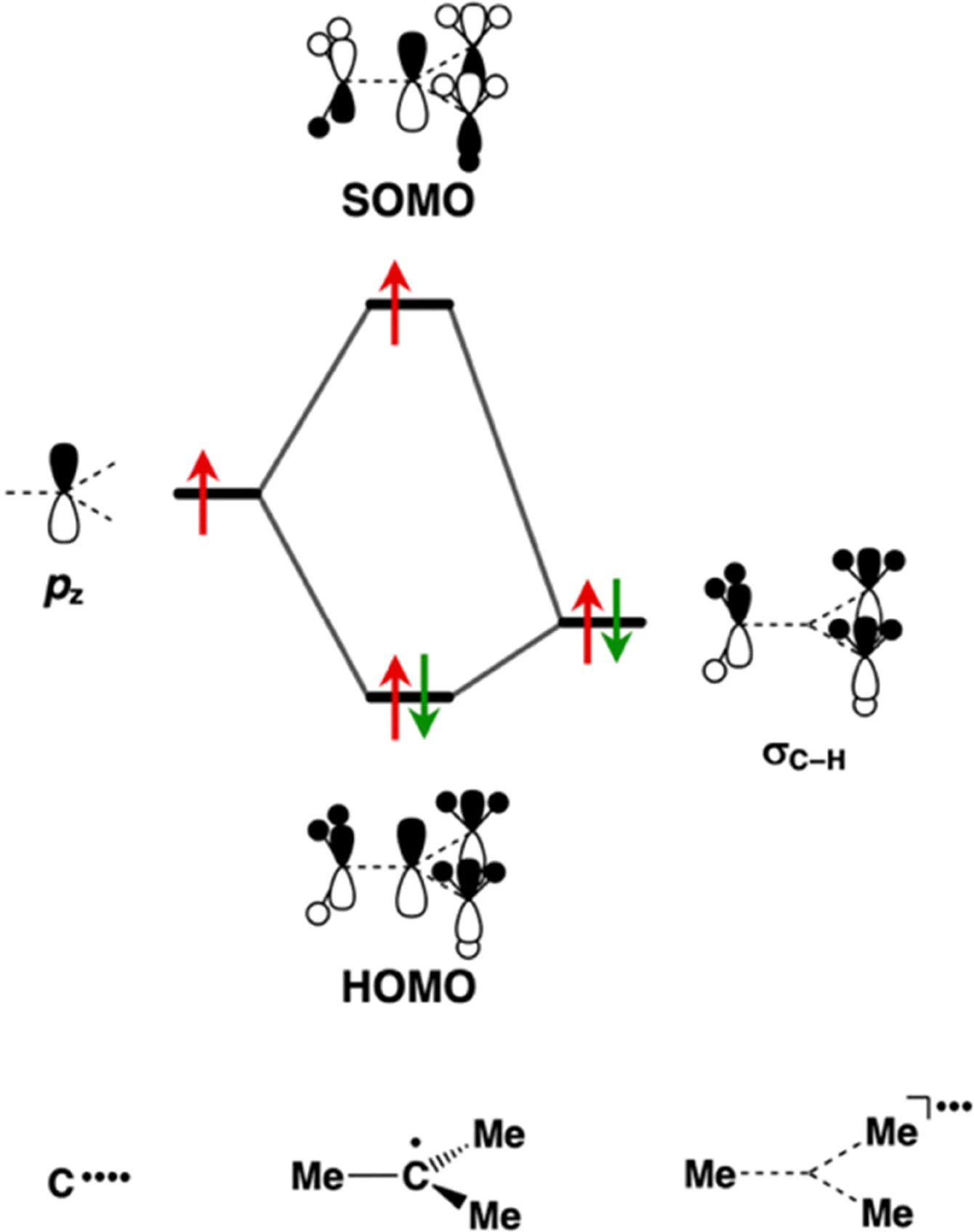
Schematic MO diagram of the *tert*-butyl radical Me_3_C˙ in terms of Me_3_˙˙˙ interacting with C˙˙˙˙ in the a_1_ irreducible representation of the *C*_3v_ symmetry. Pauli repulsion in excess spin in red and hyperconjugation in non-excess spin in green.

Herein, we reconcile the rationale through which methyl substituents influence the thermodynamic and kinetic stability of alkyl radicals. We argue that the fact that the carbon radical center is destabilized by alkyl substituents is perfectly in line with its increased nucleophilicity. To this end, we have reexamined the reactivity of alkyl radicals toward addition reactions of X_3_C˙ + Y_2_C=CY_2_ (X, Y = H, Me; Scheme 1) using the activation strain model (ASM)^9^ in conjunction with quantitative Kohn–Sham molecular orbital (KS-MO)^10^ theory and a matching energy decomposition analysis (EDA).^11^ Our results reveal that the radical addition reaction barrier decreases as the degree of methyl substitution in the radical X_3_C˙ increases, due to both polar effects and attractive steric interactions.^12^ In contrast, increasing the degree of substitution in the alkene Y_2_C=CY_2_ introduces steric hindrance, which counteracts these stabilizing interactions and raises the reaction barrier. Interestingly, we find that, in the case of a sterically crowded substrate, the steric effects counteract the electronic effects, thus deactivating the radical kinetically.

**Scheme 1:**
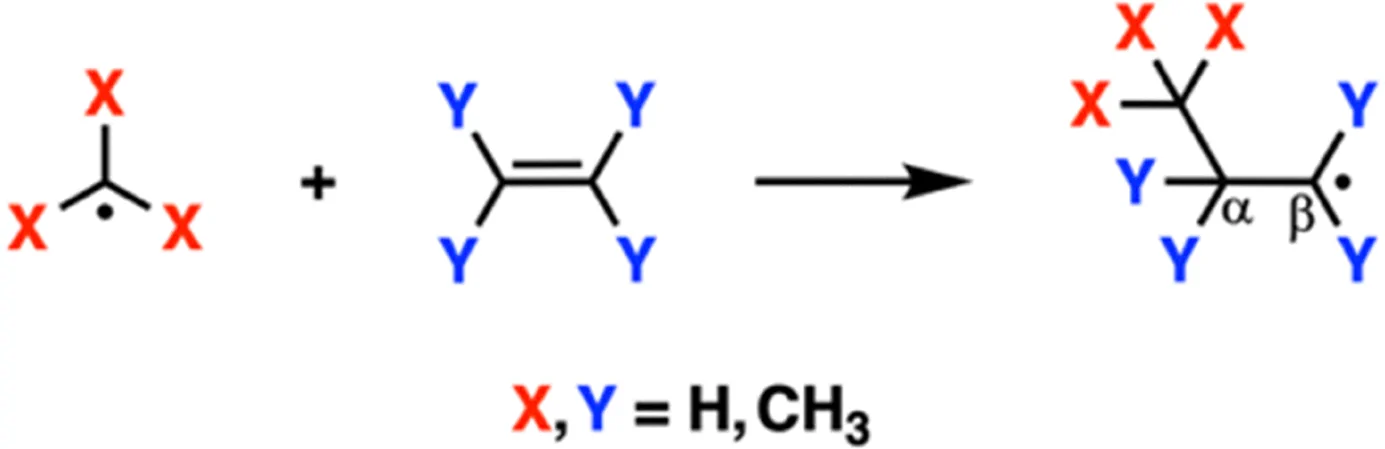
Radical addition reaction of X_3_C˙ + Y_2_C=CY_2_ (X, Y = H, Me).

Fig. 2 shows the geometries and energies of the stationary points along the potential energy surface (PES) of the model addition reactions of X_3_C˙ + Y_2_C=CY_2_ (X, Y = H, Me) computed at ZORA^13^-(U)M06-2X^14^/TZ2P^15^ using the Amsterdam Density Functional (ADF)^16^ program, as implemented in the Amsterdam Modeling Suite (AMS)^17^ software package (see Computational Methods in the SI for details).^18–20^ Energies are calculated relative to the separate reactants. The radical addition reactions proceed from the reactants (R) *via* the formation of a reactant complex (RC) toward the transition state (TS) and finally to the product (P). The RCs are weakly stabilizing, ranging from −1.1 kcal mol^−1^ for X, Y = H to −5.1 kcal mol^−1^ for X, Y = Me, and become even destabilizing with respect to the Gibbs free energies (Table S2). The TSs are early, and the reaction barriers Δ*E*^‡^ are small, up to 7.2 kcal mol^−1^, consistent with earlier reports in the literature.^7,8,21^ Note that, at the transition state, the alkene Y_2_C=CY_2_ is barely deformed from its equilibrium geometry, with a small C=C bond stretch of 0.03–0.04 Å. The radical X_3_C˙ approaches the alkene Y_2_C=CY_2_ with an angle of attack (X_3_C˙⋯C=C) of 99° to 111°, which is relatively close to the corresponding angle in the final product (109° to 115°, respectively).^22^ For the unsubstituted alkene H_2_C=CH_2_, we find that the reaction barrier Δ*E*^‡^ decreases as the degree of substitution of the radical X_3_C˙ increases (*e.g.*, for Y = H, Δ*E*^‡^ goes from 6.1 to 3.6 kcal mol^−1^ for X = H and Me, respectively).^23^ This is in line with kinetic studies that show an increased rate constant for reactions with Me_3_C˙ compared to H_3_C˙.^7,24^ On the other hand, the reaction barrier Δ*E*^‡^ rises with an increase in the degree of substitution of the alkene Y_2_C=CY_2_. For X = H, Δ*E*^‡^ goes from 6.1 to 6.9 kcal mol^−1^ for Y = H and Me, respectively, and for X = Me, Δ*E*^‡^ even goes from 3.6 to 7.2 kcal mol^−1^ for Y = H and Me, respectively. The deactivation of sterically crowded double bonds is sufficiently large to overrule the initial activation of methyl-substituted radicals, especially for larger alkyl substituents. For Y = iPr, Δ*E*^‡^ goes from 13.5 to 30.8 kcal mol^−1^ for X = H and Me, respectively (Tables S1 and S2). The trends in Δ*E*^‡^, in general, determine the trends in Δ*G*^‡^ (Table S2).

**Fig. 2:**
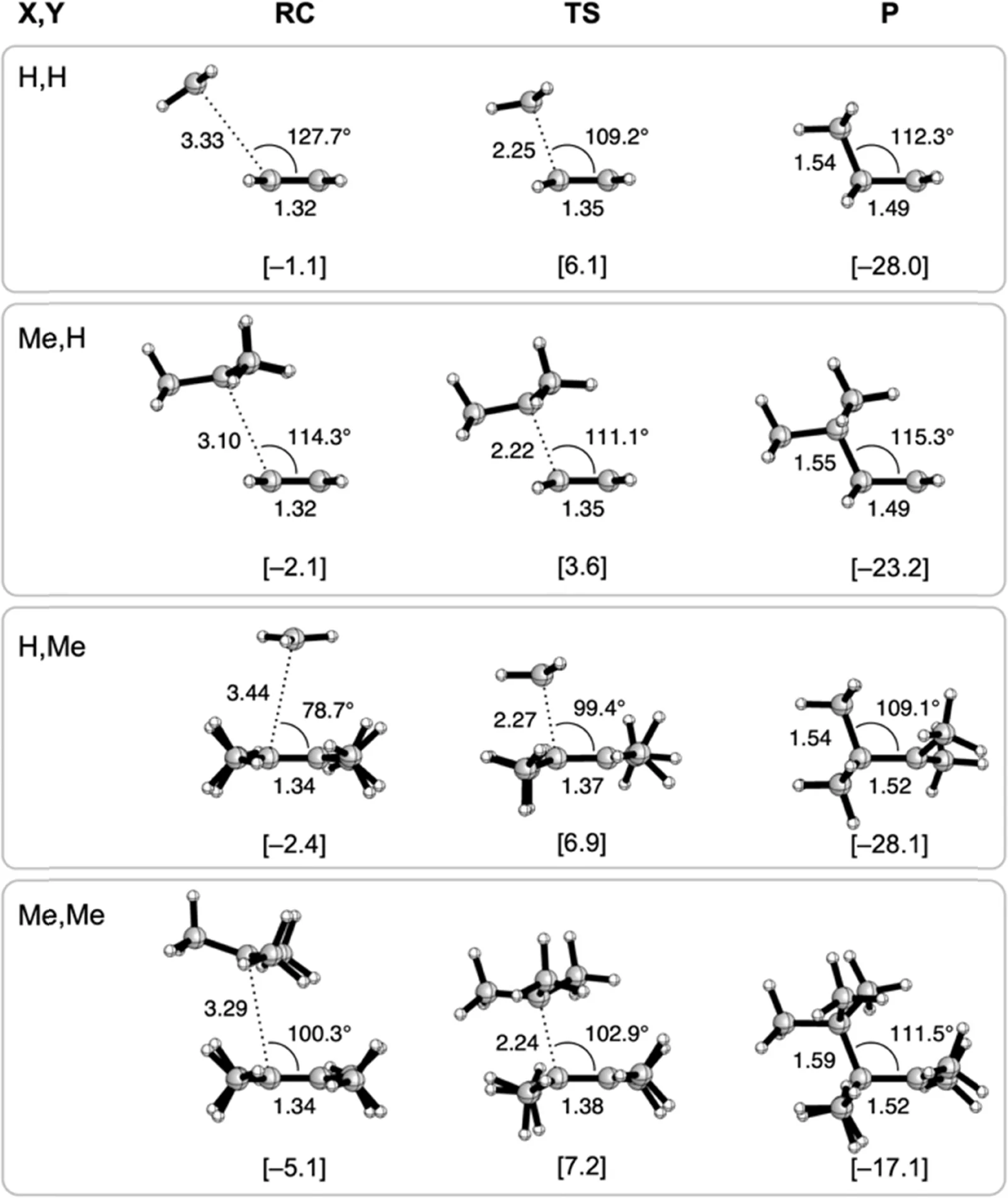
Structures (in Å, deg.) and energies (in kcal mol^−1^) of the stationary points for the radical addition reaction of X_3_C˙ + Y_2_C=CY_2_ (X, Y = H, Me) computed at ZORA-(U)M06-2X/TZ2P. RC: reactant complex, TS: transition state, and P: product.

Radical addition reactions are known to be exothermic (Table S2), as a stronger σ bond replaces a weaker π bond.^8^ Reaction energies Δ*E*_rxn_ are the most stabilizing for radical addition reactions involving the methyl radical H_3_C˙, following the order in strength of the forming C–C bond, which is stronger at primary than tertiary carbons.^3*a*,3*c*,25^ Therefore, although the reaction barrier Δ*E*^‡^ is higher for H_3_C˙ than Me_3_C˙, the reaction with the former is thermodynamically favored over the latter.

To better understand how methyl substitution affects the reaction barriers Δ*E*^‡^ and, therefore, the reactivity of alkyl radicals X_3_C˙ and alkenes Y_2_C=CY_2_, we investigate their radical addition reactions within the framework of Kohn–Sham molecular orbital (KS-MO)^10^ theory. Energies Δ*E* as a function of the newly forming C–C bond in X_3_C˙ + Y_2_C=CY_2_ (X, Y = H, Me) are depicted in Fig. 3. To gain insights into the factors driving the observed reactivity trends, we decompose Δ*E* using the activation strain model (ASM)^9^ and canonical energy decomposition analysis (EDA)^11^ (a comprehensive overview is provided in the Computational methods section of the SI). The open-shell PyFrag2019 program was used to facilitate these analyses,^26^ and the results are also shown in Fig. 3.

**Fig. 3:**
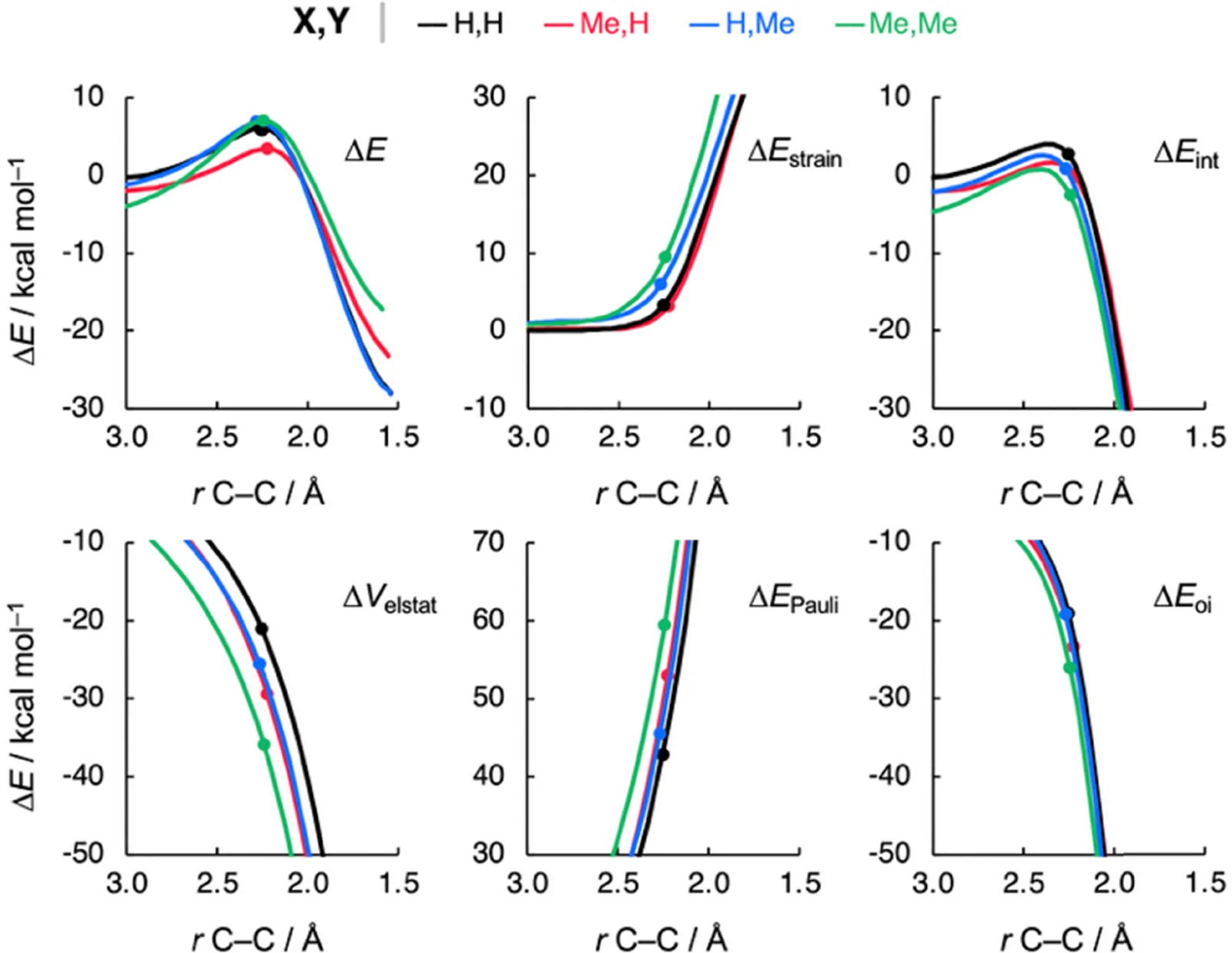
Activation strain model (ASM) and energy decomposition analysis (EDA) as a function of the newly forming C–C bond in the radical addition reaction of X_3_C˙ + Y_2_C=CY_2_ (X, Y = H, Me) computed at ZORA-(U)M06-2X/TZ2P. Transition states are indicated with a dot.

First, we examine the effect of methyl substitution on the reactivity of the radical X_3_C˙ towards the unsubstituted substrate. The decrease in reaction barrier Δ*E*^‡^ upon methyl substitution at X_3_C˙ (from black to red lines in Fig. 3) arises from a more stabilizing interaction Δ*E*_int_ energy of H_2_C=CH_2_ with Me_3_C˙ than H_3_C˙. Note that the strain energy Δ*E*_strain_ curves are nearly superimposed, reflecting a similar degree of deformation of the reactants on the course of the reaction in both cases (see Table S4 for ASM and EDA terms at a consistent geometry with a newly forming C–C bond distance of 2.25 Å; this is near all TSs, which occur between C–C distances of 2.22 and 2.27 Å). The more stabilizing Δ*E*_int_ energy for Me_3_C˙ + H_2_C=CH_2_ than H_3_C˙ + H_2_C=CH_2_, in turn, originates from more stabilizing orbital and electrostatic interactions, Δ*E*_oi_ and Δ*V*_elstat_, respectively. Methyl substitution increases the electron-donating capability, *i.e.*, the nucleophilicity, of the radical X_3_C˙, which is reflected by a larger extent of electronic charge donation from the radical to the alkene (Δ*Q*_CX3˙_ > 0 for X, Y = Me, H; Fig. 4).^27^ The reason for the enhanced nucleophilicity is that the SOMO of Me_3_C˙ is raised in energy relative to H_3_C˙, resulting in a smaller orbital energy gap Δ*ε* of the SOMO–LUMO interaction (Δ*ε*_SOMO–LUMO_ = 8.9 and 7.1 eV for X, Y = H, H and Me, H, respectively; Fig. 4). This is slightly, but not completely, offset by the increase in Δ*ε* of the HOMO–SOMO interaction (Δ*ε*_HOMO–SOMO_ = 7.8 and 8.9 eV for X, Y = H, H and Me, H, respectively). This is exactly the physical mechanism behind polar effects described in the literature.^8^ Note that methyl substitution also increases the electron-donating ability of the alkene Y_2_C=CY_2_, that is, more charge is donated to the radical from the alkene (Δ*Q*_CX3˙_ < 0 for X, Y = H, Me; Fig. 4). In this case, the HOMO of Me_2_C=CMe_2_ is raised in energy relative to H_2_C=CH_2_, leading to a smaller orbital energy gap Δε of the HOMO–SOMO interaction (Δε_HOMO–SOMO_ = 7.8 and 6.0 eV for X, Y = H, H and H, Me, respectively; Fig. 4). For X, Y = Me, Me, both SOMO–LUMO and HOMO–SOMO interactions are favored, resulting in the strongest orbital interactions Δ*E*_oi_ in our series of radical addition reactions. As the charge-transfer occurs in both directions, from and to the radical, Δ*Q*_CX3˙_ is ca 0 for X, Y = Me, Me (Fig. 4).

**Fig. 4:**
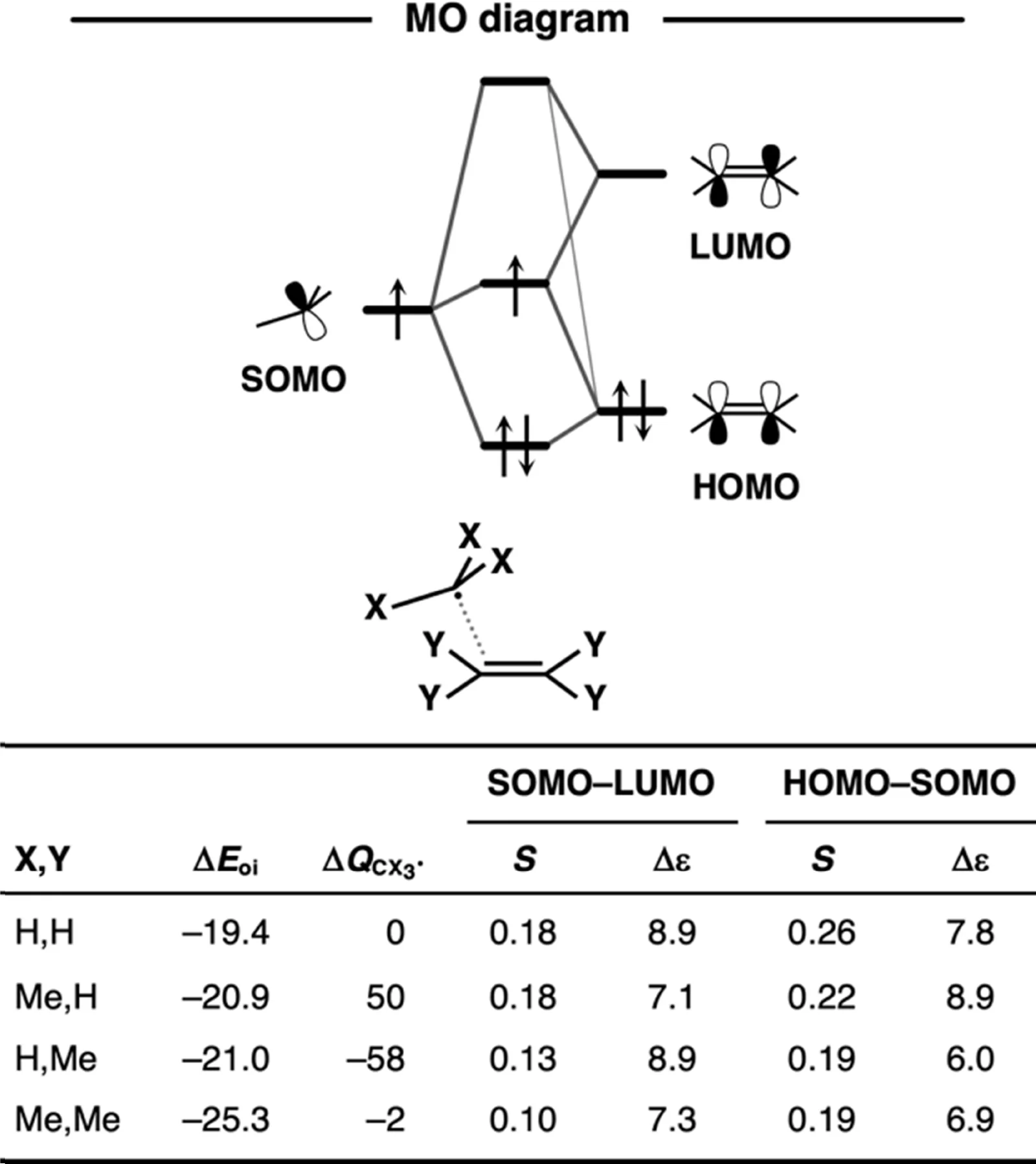
Schematic MO diagram with the orbital overlap *S* and energy gap Δε (in eV) of the interaction between the occupied SOMO spin–orbital of X_3_C˙ and the LUMO of Y_2_C=CY_2_ and between the unoccupied SOMO spin–orbital of X_3_C˙ and the HOMO of Y_2_C=CY_2_ (X, Y = H, Me), total orbital interactions Δ*E*_oi_ (in kcal mol^−1^), and Voronoi deformation density (VDD) charges of the radical Δ*Q*_CX3˙_ (in milli-electrons) associated with the formation of the C–C bond. Computed at ZORA-UM06-2X/TZ2P at the consistent geometries with a newly forming C–C bond distance of 2.25 Å.

Notably, methyl substitution increases the magnitude of stabilizing electrostatic interactions Δ*V*_elstat_, which becomes more stabilizing from H_3_C˙ to Me_3_C˙ (Fig. 3). This is in line with the general fact that, for neutral systems, the Δ*V*_elstat_ term is predominantly stabilizing and favors steric crowding,^28^ also referred to as steric attraction.^12,29^ Steric Pauli repulsion Δ*E*_Pauli_ also becomes more destabilizing upon methyl substitution. The number of closed-shell orbitals in X_3_C˙ increases as X goes from H to Me, thus providing additional destabilizing contributions to Δ*E*_Pauli_ (see Fig. S2 for the complete occupied–occupied orbital overlap matrix). The increase in Δ*E*_Pauli_, however, does not overrule the increase in Δ*E*_oi_ and Δ*V*_elstat_. As a result, the total interaction energy Δ*E*_int_ remains more stabilizing for radical addition reactions involving Me_3_C˙ than for those involving H_3_C˙. The former, therefore, goes with a smaller barrier.^30^

On the other hand, the increase in reaction barrier Δ*E*^‡^ upon methyl substitution at the alkene Y_2_C=CY_2_ (from black to blue lines in Fig. 3) does not originate from the trends in the interaction energy Δ*E*_int_, which actually become more stabilizing from H_2_C=CH_2_ to Me_2_C=CMe_2_. As discussed above, methyl substitution results in more stabilizing Δ*E*_oi_ (Fig. 4) and Δ*V*_elstat_, which is partly, but not entirely, offset by a more destabilizing Δ*E*_Pauli_ (Fig. S3). Note, however, that, in this case, part of the enhanced steric repulsion is absorbed into a more destabilizing strain Δ*E*_strain_ (Fig. 3).^3,28*b*^ The methyl-substituted alkene Me_2_C=CMe_2_ deforms more in the course of the reaction than ethylene H_2_C=CH_2_ (Δ*E*_strain,alk_ in Fig. S4). This larger deformation is mainly associated with a larger pyramidalization around the C_α_ carbon atom of the alkene (Scheme 1 and Fig. S5). That is, the methyl-substituted alkene pyramidalizes more away from the incoming radical to reduce the buildup of steric Pauli repulsion. The energy cost associated with this geometrical deformation acts as an energy penalty for the reaction to happen. Although this strain energy is smaller than the steric repulsion in the artificially not deformed alkene (Table S4), it still causes an increase in the reaction barrier as the alkene goes from H_2_C=CH_2_ to Me_2_C=CMe_2_. This steric repulsion mechanism is significant enough to override the intrinsic activation of methyl-substituted radicals, thereby decreasing the reactivity of radical additions to crowded double bonds.

In summary, methyl substituents always destabilize organic radicals thermodynamically,^3^ but they can either enhance or attenuate their reactivity (*i.e.*, make them kinetically less or more stable, respectively). Hyperconjugation raises the energy of the unpaired electron, making alkyl-substituted radicals more nucleophilic. However, this activation of methyl-substituted radicals can be mitigated or overruled by the greater steric demand of methyl (or alkyl) substituents. This happens if the alkene to which the radical is adding is sterically crowded. Overall, our findings unify the thermodynamic and kinetic effects of methyl substitution on radicals: Methyl substitution (i) destabilizes the radical thermodynamically; (ii) destabilizes the radical's unpaired electron, thus enhancing its reactivity; and (iii) aggravates the radical's steric repulsion with the alkene, reducing its reactivity.

## Author contributions

FMB and JMS conceived the project, which was supervised by FMB. DRS carried out the quantum-chemical computations and bonding analyses and drafted the manuscript. All authors discussed the results and reviewed the manuscript.

## Conflicts of interest

There are no conflicts to declare.

## Supplementary Material

CP-028-D6CP03338E-s001

## Data Availability

All data underlying this study are available in the published article and its supplementary information (SI). Supplementary information is available. See DOI: https://doi.org/10.1039/d6cp03338e.
