# Supplementary material for: Organic radical reactivity: activation and deactivation through alkyl substitution
Source: Phys Chem Chem Phys. 2026 Sep 9;28(38):23383–8. doi: 10.1039/d6cp03338e (PMC13584644; doi:10.1039/d6cp03338e)
Supplement: Supplementary file 1 [file CP-028-D6CP03338E-s001.pdf]

Supplementary Information (SI) for Physical Chemistry Chemical Physics.
This journal is © the Owner Societies 2026

Supplementary information for

Organic Radical Reactivity: Activation and Deactivation through Alkyl Substitution

Daniela Rodrigues Silva,[a] J. Martijn van der Schuur,[b] F. Matthias Bickelhaupt*[a,c,d]

a Dr. D. Rodrigues Silva, Prof. Dr. F. M. Bickelhaupt
Department of Chemistry and Pharmaceutical Sciences, AIMMS, Vrije Universiteit Amsterdam, De Boelelaan 1108, 1081 HZ Amsterdam, The Netherlands.
E–mail: f.m.bickelhaupt@vu.nl
Web address: https://www.theochem.nl

b Dr. Ir. J. M. van der Schuur
Polymer Specialties, Nouryon, Zutphenseweg 10, 7418 AJ Deventer, The Netherlands.

c Prof. Dr. F. M. Bickelhaupt
Institute of Molecules and Materials, Radboud University, Heyendaalseweg 135, 6525 AJ Nijmegen, The Netherlands.

d Prof. Dr. F. M. Bickelhaupt
Department of Chemical Sciences, University of Johannesburg, Auckland Park, Johannesburg 2006, South Africa.

Content

Methods S3

Table S1 Electronic energies ΔE (in kcal mol^{-1}) relative to the separate reactants of the stationary points of the radical addition reaction of $X_3C^\bullet + Y_2C{=}CY_2$ (X, Y = H, Me) S8

Table S2 Electronic energies ΔE, enthalpies ΔH, and Gibbs free energies ΔG (in kcal mol^{-1}) relative to the separate reactants of the stationary points of the radical addition reaction of $X_3C^\bullet + Y_2C{=}CY_2$ (X, Y = H, Me) S8

Table S3 Comparison between experimental rate constants and computed reaction barriers of the radical addition reaction of $X_3C^\bullet + H_2C{=}CHZ$ (X = H, Me; Z = $PO(OEt)_2$) S8

Table S4 Activation strain model and energy decomposition analysis terms (in kcal mol^{-1}) of the radical addition reaction of $X_3C^\bullet + Y_2C{=}CY_2$ (X, Y = H, Me) computed at a consistent geometry with a newly forming C–C bond distance of 2.25 Å S9

Fig. S1 Activation strain model (ASM) and energy decomposition analysis (EDA) as a function of the newly forming C–C bond in the radical addition reaction of $X_3C^{\bullet}$ + $Y_2C{=}CY_2$ (X, Y = H, Me) computed at ZORA-(U)OLYP-D3(BJ)/TZ2P. S9

Fig. S2 Occupied–occupied orbital overlap integrals of $X_3C^{\bullet}$ + $H_2C{=}CH_2$, wherein X = a) H and b) Me. Excess and non-excess spin are shown in plain text and italics, respectively. Computed at a consistent geometry with a newly forming C–C bond distance of 2.25 Å at ZORA-(U)M06-2X/TZ2P S10

Fig. S3 Occupied–occupied orbital overlap integrals of $H_3C^{\bullet}$ + $Y_2C{=}CY_2$, wherein Y = a) H and b) Me. Excess and non-excess spin are shown in plain text and italics, respectively. Computed at a consistent geometry with a newly forming C–C bond distance of 2.25 Å at ZORA-(U)M06-2X/TZ2P S11

Fig. S4 Strain energy terms as a function of the newly forming C–C bond in the radical addition reaction of $X_3C^{\bullet}$ + $Y_2C{=}CY_2$ (X, Y = H, Me) computed at ZORA-(U)M06-2X/TZ2P S12

Fig. S5 Key geometrical deformation as a function of the newly forming C–C bond in the radical addition reaction of $X_3C^{\bullet}$ + $Y_2C{=}CY_2$ (X, Y = H, Me) computed at ZORA-(U)M06-2X/TZ2P S12

Table S5 Cartesian coordinates (in Å), ADF total energies (in kcal mol^{-1}), and number of imaginary frequencies of the stationary points of the radical addition reactions in this study, computed at ZORA-(U)M06-2X/TZ2P. S13

Table S6 Cartesian coordinates (in Å), ADF total energies (in kcal mol^{-1}), and number of imaginary frequencies of the stationary points of the radical addition reactions in this study, computed at ZORA-(U)OLYP-D3(BJ)/TZ2P. S24

Table S7 Cartesian coordinates (in Å), ADF total energies (in kcal mol^{-1}), and number of imaginary frequencies of the stationary points of the radical addition reactions in this study, computed at ZORA-(U)OLYP/TZ2P. S30

Methods

Computational details

All calculations were performed using the Amsterdam Density Functional (ADF) program (ADF2023.1),[1] which is part of the Amsterdam Modeling Suite (AMS2023.1) software package.[2] Geometries and energies were calculated using the meta-hybrid generalized gradient approximation (GGA) M06-2X[3] functional in combination with the TZ2P[4] basis set. The TZ2P basis set is of triple-ζ quality, augmented by two sets of polarization functions, *i.e.*, 2*p* and 3*d* on H and 3*d* and 4*f* on C. All electrons were treated variationally. Scalar relativistic effects are accounted for using the zeroth-order regular approximation (ZORA).[5] The ZORA-M06-2X/TZ2P level of theory has previously been shown to agree satisfactorily with a highly correlated *ab initio* benchmark for the $H_3C^{\bullet}$ + $H_2C{=}CH_2$ model reaction, with mean absolute errors in the reaction barrier and energy of about 1 kcal mol^{-1}.[6] Herein, we have computed additional high-level relativistic CCSD(T) reference energies (see Table S1), which confirm that our DFT approach overall reproduces the reactivity trends observed at the *ab initio* level. The numerical accuracy was set to EXCELLENT.[7] Radical species were treated with a spin-unrestricted formalism. Optimized structures were confirmed to be true minima (no imaginary frequencies) or transition states (one imaginary frequency) through vibrational analyses.[8] Furthermore, the normal mode associated with the imaginary frequency was analyzed to ensure that its character corresponded to the appropriate reaction coordinate of interest. Potential energy surfaces were obtained using the intrinsic reaction coordinate (IRC) method.[9] Molecular structures were illustrated using CYLview.[10]

Activation strain model and energy decomposition analysis

The radical addition reactions of $X_3C^{\bullet}$ + $Y_2C{=}CY_2$ (X, Y = H, Me) were analyzed using the activation strain model (ASM)[11] of chemical reactivity. The ASM is a fragment-based approach to understanding the energy profile of a chemical process in terms of the original reactants. The overall bond energy ΔE is divided into the total strain and interaction energies [eqn (S1)], ΔE_{strain} and ΔE_{int}, respectively, which are projected into the reaction coordinate ξ:

$$\Delta E(\xi) = \Delta E_{\text{strain}}(\xi) + \Delta E_{\text{int}}(\xi) \qquad \text{(S1)}$$

Here, the strain energy ΔE_{strain} is the energy associated with the geometrical changes that the reactants undergo in the course of the reaction, the interaction energy ΔE_{int} is the actual energy change when the deformed reactants mutually interact, and the reaction coordinate ξ is the newly forming C–C bond.

We further analyze the interaction energy ΔE_{int} within the framework of the Kohn-Sham

molecular orbital (KS-MO) theory[12] by dissecting it using our canonical energy decomposition analysis (EDA)[13] scheme into electrostatic interactions, Pauli repulsive orbital interactions, and stabilizing orbital interactions [eqn (S2)]:

$$\Delta E_{\text{int}} = \Delta V_{\text{elstat}} + \Delta E_{\text{Pauli}} + \Delta E_{\text{oi}} \tag{S2}$$

The electrostatic energy ΔV_{elstat} corresponds to the electrostatic interactions between the unperturbed charge distribution of the fragments, which is usually attractive. The ΔV_{elstat} term can be further divided into four components [eqn (S3)]:

$$\Delta V_{\text{elstat}} = \sum_{\substack{\alpha \in A \\ \beta \in B}} \frac{Z_\alpha Z_\beta}{R_{\alpha\beta}} - \int \sum_{\alpha \in A} \frac{Z_\alpha \rho_B(r)}{|r - R_\alpha|} dr - \int \sum_{\beta \in B} \frac{Z_\beta \rho_A(r)}{|r - R_\beta|} dr + \int\int \frac{\rho_A(r_1)\rho_B(r_2)}{r_{12}} dr_1 dr_2$$
$$+ \Delta V_{\text{elstat, N–e}} + \Delta V_{\text{elstat, e–N}} + \Delta V_{\text{elstat, e–e}} \tag{S3}$$

The first term in eqn (S3) is the electrostatic repulsion between the nuclei n of reactants A and B (A = $R_3C^{\bullet}$ and B = $Y_2C{=}CY_2$), $\Delta V_{\text{elstat, N–N}}$; the second and third terms are the electrostatic attraction between the nuclei n of reactant A and the electron density ρ of reactant B, $\Delta V_{\text{elstat, N–e}}$, and vice versa, $\Delta V_{\text{elstat, e–N}}$; while the last term is the electrostatic repulsion between the electron densities ρ of reactants A and B, $\Delta V_{\text{elstat, e–e}}$.

The Pauli repulsion ΔE_{Pauli} comprises the destabilizing interactions between occupied same-spin orbitals and is responsible for any steric repulsion. The orbital interactions ΔE_{oi} term accounts for electron-pair bonding (the SOMO–SOMO interaction), charge transfer (donor–acceptor interaction between an occupied orbital of one fragment with an empty orbital of the other fragment), and polarization (empty/occupied orbital mixing on one fragment due to the presence of another fragment). The open-shell PyFrag2019 program was used to facilitate the analysis of the radical addition reactions as a function of the newly forming C–C bond.[14]

Voronoi deformation density (VDD) analysis

The flow of charge from one reactant to the other due to the chemical bond formation was analyzed using the Voronoi Deformation Density (VDD) method.[15] In the VDD method, the spatial integration of the deformation density over the Voronoi cell of atom A, a non-overlapping region of space that is closer to nucleus A than to any other nucleus, yields the VDD atomic charges (Q) of atom A in eqn (S4):

$$Q = -\int_{\text{Voronoi cell of A}} \left[\rho(\mathrm{r}) - \sum_{\mathrm{i}} \rho_{\mathrm{i}}(\mathrm{r})\right] \mathrm{dr} \qquad \text{(S4)}$$

wherein the deformation density is the density change going from a superposition of the spherical atomic densities at the positions of the molecule, the so-called promolecule, to the actual density of that molecule. The physical interpretation is, therefore, straightforward: $Q > 0$ corresponds to the loss of electrons, whereas $Q < 0$ is associated with the gain of electrons in the Voronoi cell of atom A.

The VDD method can also be used to compute the charge change (ΔQ) due to chemical bond formation between molecular fragments, for example, the C–C bond formation in radical addition reactions of $X_3C^{\bullet} + Y_2C{=}CY_2$ (X, Y = H, Me). The charge change ΔQ in eqn (S5) is the final density of the overall complex $\rho_{\mathrm{complex}}(\mathrm{r})$ relative to the sum of the fragment (or reactant) densities $\rho_{\mathrm{i}}(\mathrm{r})$. Therefore, ΔQ represents the amount of electronic charge that flows into ($\Delta Q < 0$) or out of ($\Delta Q > 0$) the Voronoi cell of an atom due to the interaction between the reactants.

$$\Delta Q = -\int_{\text{Voronoi cell of A in molecule}} \left[\rho_{\mathrm{complex}}(\mathrm{r}) - \sum_{\text{fragments, i}} \rho_{\mathrm{i}}(\mathrm{r})\right] \mathrm{dr} \qquad \text{(S5)}$$

References

1 (a) ADF 2023.1; SCM: Theoretical Chemistry, Vrije Universiteit, Amsterdam, The Netherlands,, 2023, http://www.scm.com; (b) G. te Velde, F. M. Bickelhaupt, E. J. Baerends, C. Fonseca Guerra, S. J. A. van Gisbergen, J. G. Snijders and T. Ziegler, Chemistry with ADF, *J. Comput. Chem.*, 2001, **22**, 931–967; (c) C. Fonseca Guerra, J. G. Snijders, G. te Velde and E. J. Baerends, Towards an order-N DFT method, *Theor. Chem. Acc.*, 1998, **99**, 391–403.

2 (a) AMS 2023.1; SCM: Theoretical Chemistry, Vrije Universiteit, Amsterdam, The Netherlands, 2023, http://www.scm.com; (b) E. J. Baerends, N. F. Aguirre, N. D. Austin, J. Autschbach, F. M. Bickelhaupt, R. Bulo, C. Cappelli, A. C. T. van Duin, F. Egidi, C. Fonseca Guerra, A. Förster, M. Franchini, F. T. P. M. Goumans, T. Heine, M. Hellström, C. R. Jacob, L. Jensen, M. Krykunov, E. van Lenthe, A. Michalak, M. M. Mitoraj, J. Neugebauer, V. P. Nicu, P. Philipsen, H. Ramanantoanina, R. Rüger, G. Schreckenbach, M. Stener, M. Swart, J. M. Thijssen, T. Trnka, L. Visscher, A. Yakovlev, S. J. A. van Gisbergen, The Amsterdam Modeling Suite, *J. Chem. Phys.*, 2025, **162**, 162501/1–11.

3 Y. Zhao and D. G. Truhlar, The M06 suite of density functionals for main group

thermochemistry, thermochemical kinetics, noncovalent interactions, excited states, and transition elements: two new functionals and systematic testing of four M06-class functionals and 12 other functionals, *Theor. Chem. Acc.*, 2008, **120**, 215–241.

4 E. van Lenthe and E. J. Baerends, Optimized Slater-type basis sets for the elements 1–118, *J. Comput. Chem.*, 2003, **24**, 1142–1156.

5 (a) E. van Lenthe, R. van Leeuwen, E. J. Baerends and J. G. Snijders, Relativistic regular two-component Hamiltonians, *Int. J. Quantum Chem.*, 1996, **57**, 281–293; (b) E. van Lenthe, E. J. Baerends and J. G. Snijders, Relativistic total energy using regular approximations, *J. Chem. Phys.*, 1994, **101**, 9783–9792.

6 Y. Hordijk, M. Dalla Tiezza, D. Rodrigues Silva and T. A. Hamlin, Radical Addition Reactions: Hierarchical *Ab initio* Benchmark and DFT Performance Study, *ChemPhysChem*, 2025, **26**, e202400728.

7 (a) M. Franchini, P. H. T. Philipsen, E. van Lenthe and L. Visscher, Accurate Coulomb Potentials for Periodic and Molecular Systems through Density Fitting, *J. Chem. Theory Comput.*, 2014, **10**, 1994–2004; (b) M. Franchini, P. H. T. Philipsen and L. Visscher, The Becke Fuzzy Cells Integration Scheme in the Amsterdam Density Functional Program Suite, *J. Comput. Chem.*, 2013, **34**, 1819–1827.

8 (a) A. Bérces, R. M. Dickson, L. Fan, H. Jacobsen, D. Swerhone and T. Ziegler, An implementation of the coupled perturbed Kohn-Sham equations: perturbation due to nuclear displacements, *Comput. Phys. Commun.*, 1997, **100**, 247–262; (b) H. Jacobsen, A. Bérces, D. P. Swerhone and T. Ziegler, Analytic second derivatives of molecular energies: a density functional implementation, *Comput. Phys. Commun.*, 1997, **100**, 263–276; (c) S. K. Wolff, Analytical second derivatives in the Amsterdam density functional package, *Int. J. Quantum Chem.*, 2005, **104**, 645–659.

9 (a) K. Fukui, The path of chemical reactions - the IRC approach, *Acc. Chem. Res.*, 1981, **14**, 363–368; (b) L. Deng, T. Ziegler and L. Fan, A combined density functional and intrinsic reaction coordinate study on the ground state energy surface of H_2CO, *J. Chem. Phys.*, 1993, **99**, 3823–3835; (c) L. Deng and T. Ziegler, The determination of intrinsic reaction coordinates by density functional theory, *Int. J. Quantum Chem.*, 1994, **52**, 731–765.

10 C. Y. Legault, CYLview20; Université de Sherbrooke: Sherbrooke, Quebec, Canada, 2020, www.cylview.org.

11 (a) P. Vermeeren, T. A. Hamlin and F. M. Bickelhaupt, Chemical reactivity from an activation strain perspective, *Chem. Commun.*, 2021, **57**, 5880–5896; (b) P. Vermeeren, S. C. C. van der Lubbe, C. Fonseca Guerra, F. M. Bickelhaupt and T. A. Hamlin, Understanding chemical

reactivity using the activation strain model, *Nat. Protoc.*, 2020, **15**, 649–667; (c) F. M. Bickelhaupt and K. N. Houk, Analyzing Reaction Rates with the Distortion/Interaction-Activation Strain Model, *Angew. Chem. Int. Ed.*, 2017, **56**, 10070–10086; *Angew. Chem.*, 2017, **129**, 10204–10221.

12 (a) R. van Meer, O. V. Gritsenko and E. J. Baerends, Physical Meaning of Virtual Kohn–Sham Orbitals and Orbital Energies: An Ideal Basis for the Description of Molecular Excitations, *J. Chem Theory Comput.*, 2014, **10**, 4432–4441; (b) T. A. Albright, J. K. Burdett and W. H. Wangbo, *Orbital Interactions in Chemistry*, Wiley, New York, 2013.

13 F. M. Bickelhaupt and E. J. Baerends, In *Reviews in Computational Chemistry*, ed. K. B. Lipkowitz and D. B. Boyd, Wiley–VCH, Hoboken, 2000, pp. 1–86.

14 (a) W. -J. van Zeist, C. Fonseca Guerra and F. M. Bickelhaupt, PyFrag—Streamlining your reaction path analysis, *J. Comp. Chem.*, 2008, **29**, 312–315; (b) X. Sun, T. M. Soini, J. Poater, T. A. Hamlin and F. M Bickelhaupt, PyFrag 2019—Automating the exploration and analysis of reaction mechanisms, *J. Comput. Chem.*, 2019, **40**, 2227–2233; (c) X. Sun, T. M. Soini, L. P. Wolters, W. -J. van Zeist, C. Fonseca Guerra, T. A. Hamlin and F. M. Bickelhaupt, PyFrag 2019, Vrije Universiteit Amsterdam, The Netherlands.

15 (a) C. Fonseca Guerra, J. -W. Handgraaf, E. J. Baerends and F. M. Bickelhaupt, Voronoi deformation density (VDD) charges: Assessment of the Mulliken, Bader, Hirshfeld, Weinhold, and VDD methods for charge analysis, *J. Comput. Chem.*, 2004, **25**, 189–210; (b) C. Nieuwland, P. Vermeeren, F. M. Bickelhaupt and C. Fonseca Guerra, Understanding chemistry with the symmetry-decomposed Voronoi deformation density charge analysis, *J. Comput. Chem.*, 2023, **44**, 2108–2119.

Table S1 Electronic energies Δ*E* (in kcal mol^{-1}) relative to the separate reactants of the stationary points[a] of the radical addition reaction of $X_3C^\bullet + Y_2C=CY_2$ (X, Y = H, Me, iPr).[b]

	**ZORA-(U)M06-2X/TZ2P**			**ZORA-(U)OLYP/TZ2P**			**ZORA-(U)OLYP-D3BJ/TZ2P**		
**Systems**	ΔE_{RC}	ΔE_{TS}	ΔE_{P}	ΔE_{RC}	ΔE_{TS}	ΔE_{P}	ΔE_{RC}	ΔE_{TS}	ΔE_{P}
**$H_3C^\bullet + H_2C=CH_2$**	–1.1	6.1	–28.0	–	7.5	–22.0	–	1.7	–28.8
	(–0.1)	(6.4)	(–26.1)		(6.2)	(–25.9)		(5.2)	(–25.9)
**$Me_3C^\bullet + H_2C=CH_2$**	–2.1	3.6	–23.2	–	11.2	–7.3	–2.2	–1.2	–21.6
	(–1.4)	(3.3)	(–23.1)		(3.6)	(–22.8)	(–1.3)	(2.6)	(–23.1)
**$H_3C^\bullet + Me_2C=CMe_2$**	–2.4	6.9	–28.1	–	11.8	–16.8	–	1.2	–28.8
	(–0.9)	(6.6)	(–27.0)		(7.0)	(–26.4)		(5.7)	(–26.9)
**$Me_3C^\bullet + Me_2C=CMe_2$**	–5.1	7.2	–17.1	–	24.1	10.3	–4.8	0.8	–15.7
	(–4.1)	(5.7)	(–18.3)		(7.1)	(–17.3)	(–4.3)	(5.5)	(–18.1)
**$H_3C^\bullet + iPr_2C=CiPr_2$**	–2.2	13.5	–20.1	–	26.3	0.5	–	8.4	–18.3
**$Me_3C^\bullet + iPr_2C=CiPr_2$**	–3.6	30.9	15.0	–	62.5	54.6	–4.7	24.8	17.4

[a] RC: reactant complex, TS: transition state, and P: product. [b] Single-point energies at ZORA-(U)CCSD(T)/ma-ZORA-def2-TZVPP//ZORA-(U)DFT/TZ2P (DFT = M06-2X, OLYP, and OLYP-D3(BJ)) are shown in parentheses. Quasi-restricted orbitals (QROs) were used for open-shell CCSD(T) calculations.

Table S2 Electronic energies Δ*E*, enthalpies Δ*H*, and Gibbs free energies Δ*G* (in kcal mol^{-1}) relative to the separate reactants of the stationary points[a] along with the rate constants *k* (in) of the radical addition reaction of $X_3C^\bullet$ + $Y_2C=CY_2$ (X, Y = H, Me, iPr).[b]

	**RC**			**TS**				**P**		
**Systems**	**Δ*E***	**Δ*H***	**Δ*G***	**Δ*E***	**Δ*H***	**Δ*G***	***k*[c]**	**Δ*E***	**Δ*H***	**Δ*G***
**$H_3C^\bullet + H_2C=CH_2$**	–1.1	–0.5	4.4	6.1	7.3	15.9	$1.4\ 10^{1}$	–28.0	–24.5	–15.6
**$Me_3C^\bullet + H_2C=CH_2$**	–2.1	–1.1	4.5	3.6	4.0	15.1	$5.3\ 10^{1}$	–23.2	–21.2	–9.3
**$H_3C^\bullet + Me_2C=CMe_2$**	–2.4	–1.9	5.4	6.9	7.8	17.7	$6.6\ 10^{-1}$	–28.1	–24.5	–12.6
**$Me_3C^\bullet + Me_2C=CMe_2$**	–5.1	–4.6	6.9	7.2	7.7	22.7	$1.4\ 10^{-4}$	–17.1	–14.5	1.8
**$H_3C^\bullet + iPr_2C=CiPr_2$**	–2.2	–1.0	4.5	13.5	15.0	27.2	$7.1\ 10^{-8}$	–20.1	–15.8	–2.2
**$Me_3C^\bullet + iPr_2C=CiPr_2$**	–3.6	–2.4	6.6	30.9	32.8	49.9	$1.6\ 10^{-24}$	15.0	19.1	37.2

[a] RC: reactant complex, TS: transition state, and P: product. [b] Computed at ZORA-(U)M06-2X/TZ2P. [c] Rates constants were estimated using the Eyring equation, assuming a transmission coefficient of unity (κ = 1).

Table S3 Comparison between experimental rate constants[a] and computed reaction barriers[b] of the radical addition reaction of $X_3C^\bullet + H_2C=CHZ$ (X = H, Me; Z = $PO(OEt)_2$).

**Systems**	***k* / L mol^{-1} s^{-1}**	**$\Delta E^{‡}$/ kcal mol^{-1}**
**$H_3C^\bullet + H_2C=CHPO(OEt)_2$**	$2.5\ 10^{3}$	6.5
**$MeH_2C^\bullet + H_2C=CHPO(OEt)_2$**	$2.6\ 10^{3}$	5.7
**$Me_2HC^\bullet + H_2C=CHPO(OEt)_2$**	$1.2\ 10^{4}$	3.8
**$Me_3C^\bullet + H_2C=CHPO(OEt)_2$**	$5.9\ 10^{4}$	2.3

[a] Rate constant at –40°C taken from ref. [7f]. [b] Computed at ZORA-(U)M06-2X/TZ2P.

Table S4 Activation strain model and energy decomposition analysis terms (in kcal mol^{-1}) of the radical addition reaction of $X_3C^{\bullet}$ + $Y_2C{=}CY_2$ (X, Y = H, Me) computed at a consistent geometry with a newly forming C–C bond distance of 2.25 Å.

Systems	ΔE	ΔE_{strain}	$\Delta E_{strain, alk}$	$\Delta E_{strain, rad}$	ΔE_{int}	ΔV_{elstat}	ΔE_{Pauli}	ΔE_{oi}
**Consistent geometry with *r* C–C = 2.25 Å**								
$H_3C^{\bullet}$ + $H_2C{=}CH_2$	6.0	3.4	2.3	1.1	2.6	–21.3	43.3	–19.4
$Me_3C^{\bullet}$ + $H_2C{=}CH_2$	3.4	2.6	2.2	0.4	0.8	–27.6	49.3	–20.9
$H_3C^{\bullet}$ + $Me_2C{=}CMe_2$	6.8	6.7	5.3	1.4	0.1	–26.6	47.7	–21.0
$Me_3C^{\bullet}$ + $Me_2C{=}CMe_2$	7.0	9.2	7.2	2.0	–2.2	–35.3	58.4	–25.3
**Consistent geometry with *r* C–C = 2.25 Å and Cα pyr = 360°**								
$H_3C^{\bullet}$ + $H_2C{=}CH_2$	8.4	2.0	0.8	1.1	6.4	–22.0	45.7	–17.2
$Me_3C^{\bullet}$ + $H_2C{=}CH_2$	5.8	1.2	0.8	0.4	4.6	–28.6	52.5	–19.3
$H_3C^{\bullet}$ + $Me_2C{=}CMe_2$	17.1	4.8	3.4	1.4	12.3	–31.8	62.4	–18.4
$Me_3C^{\bullet}$ + $Me_2C{=}CMe_2$	44.4	7.1	5.1	2.0	37.3	–59.4	127.1	–30.4
**Consistent geometry with *r* C–C = 1.59 Å**								
$H_3C^{\bullet}$ + $H_2C{=}CH_2$	–26.7	41.3	33.8	7.5	–68.0	–114.4	254.2	–207.8
$Me_3C^{\bullet}$ + $H_2C{=}CH_2$	–22.0	42.7	33.7	9.0	–64.7	–135.8	277.8	–206.7
$H_3C^{\bullet}$ + $Me_2C{=}CMe_2$	–26.9	47.2	39.6	7.6	–74.1	–134.7	275.3	–214.7
$Me_3C^{\bullet}$ + $Me_2C{=}CMe_2$	–17.0	53.5	42.6	10.9	–70.5	–161.0	311.7	–221.2

[a] Computed at ZORA-(U)M06-2X/TZ2P.

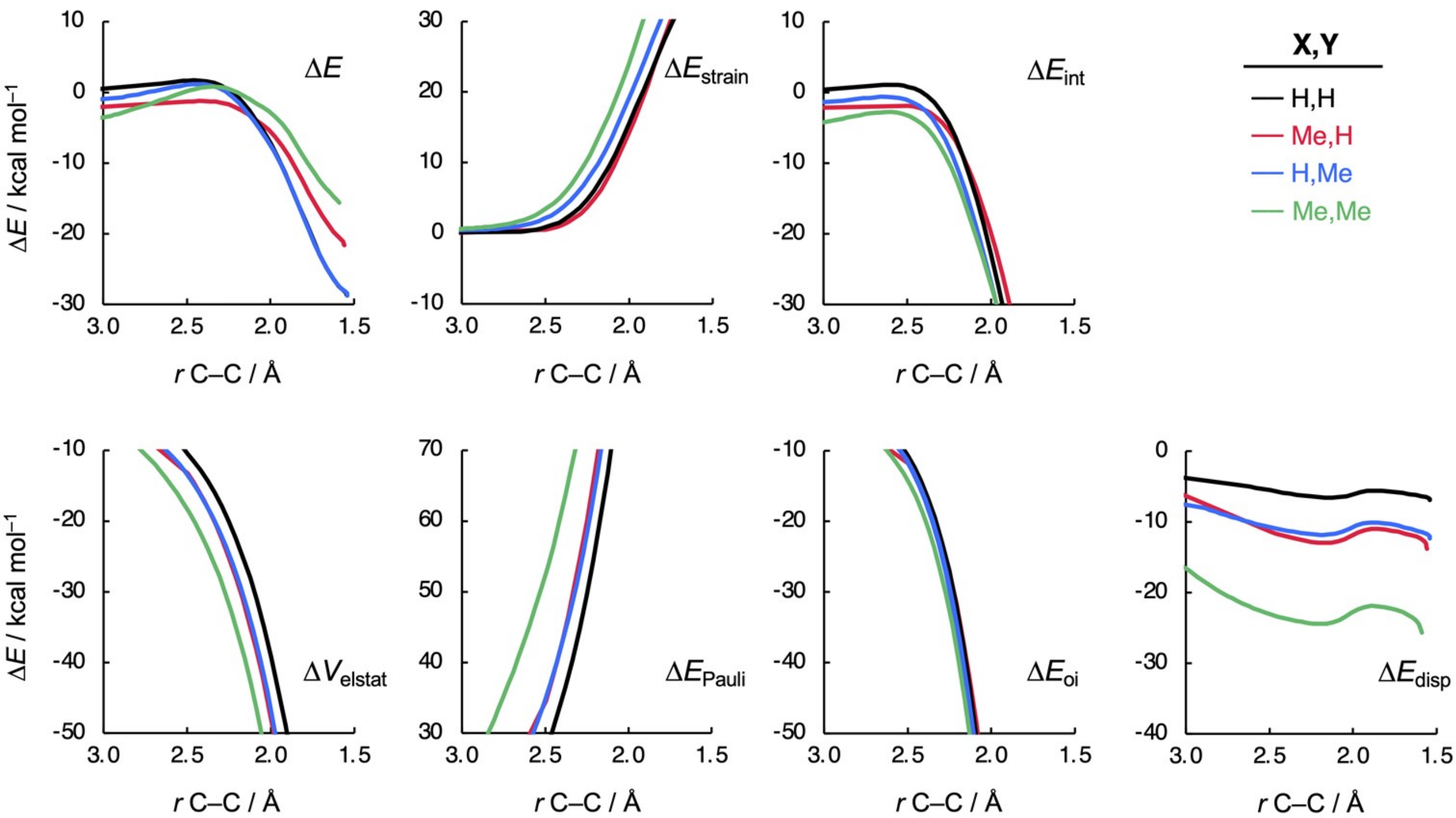

Fig. S1 Activation strain model (ASM) and energy decomposition analysis (EDA) as a function of the newly forming C–C bond in the radical addition reaction of $X_3C^{\bullet}$ + $Y_2C{=}CY_2$ (X, Y = H, Me) computed at ZORA-(U)OLYP-D3(BJ)/TZ2P.

a)

$CH_3^•$ \ $H_2C=CH_2$	1A	2A	3A	4A	5A	6A	7A	8A
4A	0.00 / *0.00*	0.00 / *0.00*	0.00 / *0.00*	0.00 / *0.00*	0.04 / *0.05*	0.00 / *0.00*	0.03 / *0.03*	0.00 / *0.00*
3A	0.00 / *0.00*	0.00 / *0.00*	0.01 / *0.01*	0.03 / *0.03*	0.00 / *0.00*	0.03 / *0.03*	0.00 / *0.00*	0.03 / *0.03*
2A	0.00 / *0.00*	0.00 / *0.00*	0.06 / *0.06*	0.05 / *0.05*	0.00 / *0.00*	0.03 / *0.03*	0.00 / *0.00*	0.15 / *0.14*
1A	0.00 / *0.00*	0.00 / *0.00*	0.00 / *0.00*	0.00 / *0.00*	0.00 / *0.00*	0.00 / *0.00*	0.00 / *0.00*	0.02 / *0.02*

b)

$CMe_3^•$ \ $H_2C=CH_2$	1A	2A	3A	4A	5A	6A	7A	8A
16A	0.00 / *0.00*	0.00 / *0.00*	0.00 / *0.00*	0.00 / *0.00*	0.01 / *0.00*	0.00 / *0.00*	0.01 / *0.0*	0.00 / *0.00*
15A	0.00 / *0.00*	0.00 / *0.00*	0.01 / *0.01*	0.01 / *0.01*	0.00 / *0.00*	0.01 / *0.01*	0.00 / *0.00*	0.02 / *0.02*
14A	0.00 / *0.00*	0.00 / *0.00*	0.00 / *0.00*	0.00 / *0.00*	0.01 / *0.01*	0.00 / *0.00*	0.01 / *0.01*	0.00 / *0.00*
13A	0.00 / *0.00*	0.00 / *0.00*	0.00 / *0.00*	0.00 / *0.00*	0.04 / *0.05*	0.00 / *0.00*	0.01 / *0.01*	0.00 / *0.00*
12A	0.00 / *0.00*	0.00 / *0.00*	0.01 / *0.01*	0.02 / *0.03*	0.00 / *0.00*	0.03 / *0.03*	0.00 / *0.00*	0.02 / *0.02*
11A	0.00 / *0.00*	0.00 / *0.00*	0.00 / *0.00*	0.00 / *0.00*	0.03 / *0.02*	0.00 / *0.00*	0.05 / *0.05*	0.00 / *0.00*
10A	0.00 / *0.00*	0.00 / *0.00*	0.01 / *0.01*	0.03 / *0.02*	0.00 / *0.00*	0.02 / *0.02*	0.00 / *0.00*	0.03 / *0.03*
9A	0.00 / *0.00*	0.01 / *0.01*	0.08 / *0.07*	0.06 / *0.05*	0.00 / *0.00*	0.04 / *0.04*	0.00 / *0.00*	0.14 / *0.12*
8A	0.00 / *0.00*	0.00 / *0.00*	0.03 / *0.01*	0.03 / *0.02*	0.00 / *0.00*	0.01 / *0.00*	0.00 / *0.00*	0.04 / *0.03*
7A	0.00 / *0.00*	0.00 / *0.00*	0.00 / *0.00*	0.00 / *0.00*	0.03 / *0.03*	0.00 / *0.00*	0.02 / *0.02*	0.00 / *0.00*
6A	0.00 / *0.00*	0.00 / *0.00*	0.00 / *0.00*	0.02 / *0.02*	0.00 / *0.00*	0.02 / *0.02*	0.00 / *0.00*	0.03 / *0.03*
5A	0.00 / *0.00*	0.00 / *0.00*	0.05 / *0.04*	0.05 / *0.04*	0.00 / *0.00*	0.03 / *0.03*	0.00 / *0.00*	0.14 / *0.13*
4A	0.00 / *0.00*	0.00 / *0.00*	0.00 / *0.00*	0.00 / *0.00*	0.00 / *0.00*	0.00 / *0.00*	0.00 / *0.00*	0.01 / *0.01*
3A	0.00 / *0.00*	0.00 / *0.00*	0.00 / *0.00*	0.00 / *0.00*	0.00 / *0.00*	0.00 / *0.00*	0.00 / *0.00*	0.00 / *0.00*
2A	0.00 / *0.00*	0.00 / *0.00*	0.00 / *0.00*	0.00 / *0.00*	0.00 / *0.00*	0.00 / *0.00*	0.00 / *0.00*	0.00 / *0.00*
1A	0.00 / *0.00*	0.00 / *0.00*	0.00 / *0.00*	0.00 / *0.00*	0.00 / *0.00*	0.00 / *0.00*	0.00 / *0.00*	0.02 / *0.02*

Fig. S2 Occupied–occupied orbital overlap integrals of $X_3C^•$ + $H_2C=CH_2$, wherein X = a) H and b) Me. Excess and non-excess spin are shown in plain text and italics, respectively. Computed at a consistent geometry with a newly forming C–C bond distance of 2.25 Å at ZORA-(U)M06-2X/TZ2P.

a)

$H_2C{=}CH_2$ \ $CH_3^•$	1A	2A	3A	4A
8A	0.02<br>*0.02*	0.15<br>*0.14*	0.03<br>*0.03*	0.00<br>*0.00*
7A	0.00<br>*0.00*	0.00<br>*0.00*	0.00<br>*0.00*	0.03<br>*0.03*
6A	0.00<br>*0.00*	0.03<br>*0.03*	0.03<br>*0.03*	0.00<br>*0.00*
5A	0.00<br>*0.00*	0.00<br>*0.00*	0.00<br>*0.00*	0.04<br>*0.05*
4A	0.00<br>*0.00*	0.05<br>*0.05*	0.03<br>*0.03*	0.00<br>*0.00*
3A	0.00<br>*0.00*	0.06<br>*0.06*	0.01<br>*0.01*	0.00<br>*0.00*
2A	0.00<br>*0.00*	0.00<br>*0.00*	0.00<br>*0.00*	0.00<br>*0.00*
1A	0.00<br>*0.00*	0.00<br>*0.00*	0.00<br>*0.00*	0.00<br>*0.00*

b)

$Me_2C{=}CMe_2$ \ $CH_3^•$	1A	2A	3A	4A
24A	0.02<br>*0.02*	0.13<br>*0.12*	0.01<br>*0.01*	0.04<br>*0.04*
23A	0.00<br>*0.00*	0.00<br>*0.00*	0.01<br>*0.01*	0.00<br>*0.00*
22A	0.00<br>*0.00*	0.00<br>*0.00*	0.00<br>*0.00*	0.01<br>*0.01*
21A	0.00<br>*0.00*	0.00<br>*0.00*	0.01<br>*0.01*	0.00<br>*0.00*
20A	0.00<br>*0.00*	0.00<br>*0.00*	0.00<br>*0.00*	0.00<br>*0.00*
19A	0.00<br>*0.00*	0.00<br>*0.00*	0.02<br>*0.02*	0.00<br>*0.00*
18A	0.00<br>*0.00*	0.01<br>*0.01*	0.03<br>*0.03*	0.01<br>*0.01*
17A	0.00<br>*0.00*	0.00<br>*0.00*	0.04<br>*0.04*	0.02<br>*0.02*
16A	0.00<br>*0.00*	0.04<br>*0.03*	0.00<br>*0.00*	0.04<br>*0.05*
15A	0.00<br>*0.00*	0.00<br>*0.00*	0.02<br>*0.02*	0.01<br>*0.01*
14A	0.01<br>*0.01*	0.09<br>*0.09*	0.00<br>*0.01*	0.00<br>*0.00*
13A	0.00<br>*0.00*	0.00<br>*0.00*	0.01<br>*0.01*	0.03<br>*0.03*
12A	0.00<br>*0.00*	0.02<br>*0.02*	0.00<br>*0.00*	0.00<br>*0.00*
11A	0.00<br>*0.00*	0.01<br>*0.01*	0.01<br>*0.01*	0.02<br>*0.02*
10A	0.00<br>*0.00*	0.00<br>*0.00*	0.02<br>*0.02*	0.00<br>*0.00*
9A	0.00<br>*0.00*	0.00<br>*0.00*	0.04<br>*0.04*	0.01<br>*0.01*
8A	0.00<br>*0.00*	0.03<br>*0.03*	0.01<br>*0.01*	0.03<br>*0.03*
7A	0.00<br>*0.00*	0.06<br>*0.06*	0.00<br>*0.00*	0.01<br>*0.01*
6A	0.00<br>*0.00*	0.00<br>*0.00*	0.00<br>*0.00*	0.00<br>*0.00*
5A	0.00<br>*0.00*	0.00<br>*0.00*	0.00<br>*0.00*	0.00<br>*0.00*
4A	0.00<br>*0.00*	0.00<br>*0.00*	0.00<br>*0.00*	0.00<br>*0.00*
3A	0.00<br>*0.00*	0.00<br>*0.00*	0.00<br>*0.00*	0.00<br>*0.00*
2A	0.00<br>*0.00*	0.00<br>*0.00*	0.00<br>*0.00*	0.00<br>*0.00*
1A	0.00<br>*0.00*	0.00<br>*0.00*	0.00<br>*0.00*	0.00<br>*0.00*

Fig. S3 Occupied–occupied orbital overlap integrals of $H_3C^• + Y_2C{=}CY_2$, wherein Y = a) H and b) Me. Excess and non-excess spin are shown in plain text and italics, respectively. Computed at a consistent geometry with a newly forming C–C bond distance of 2.25 Å at ZORA-(U)M06-2X/TZ2P.

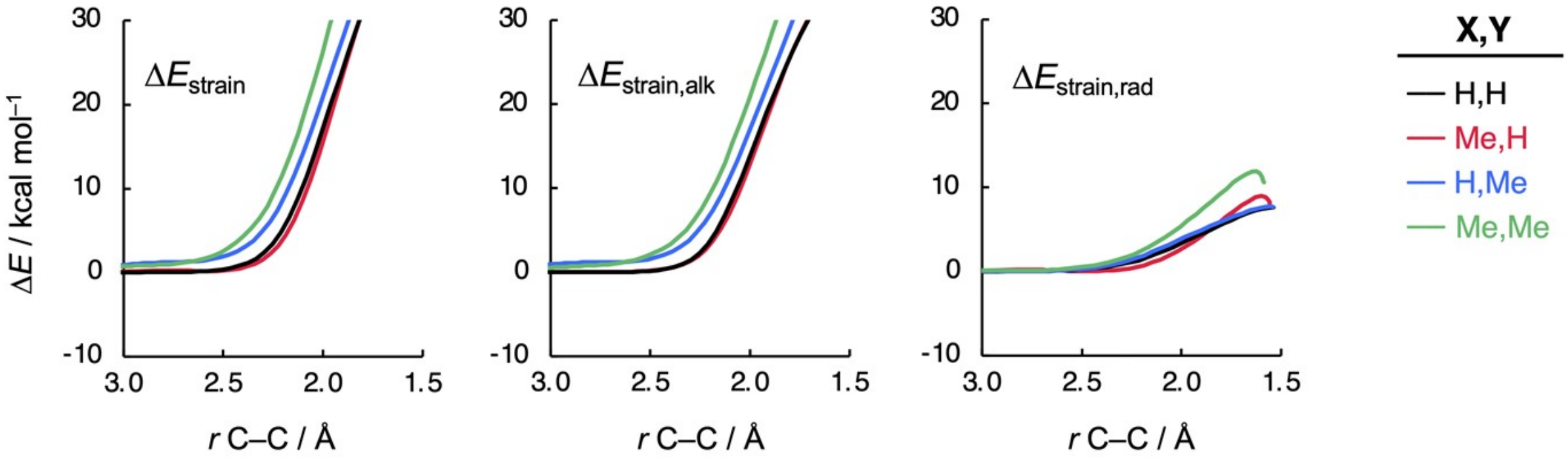

Fig. S4 Strain energy terms as a function of the newly forming C–C bond in the radical addition reaction of $X_3C^{\bullet}$ + $Y_2C{=}CY_2$ (X, Y = H, Me) computed at ZORA-(U)M06-2X/TZ2P.

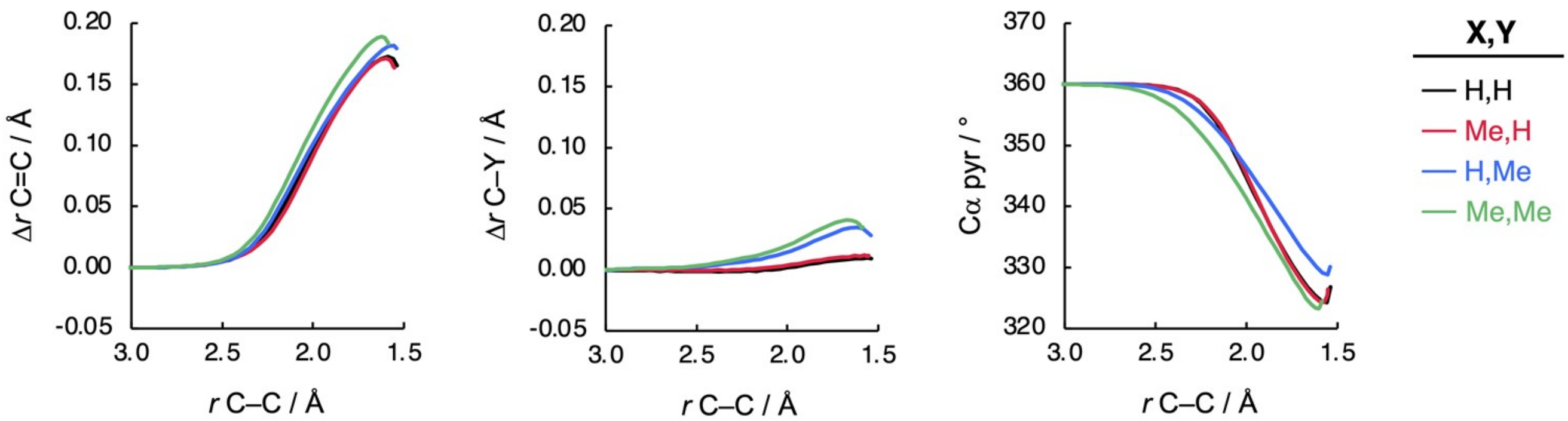

Fig. S5 Key geometrical deformation as a function of the newly forming C–C bond in the radical addition reaction of $X_3C^{\bullet}$ + $Y_2C{=}CY_2$ (X, Y = H, Me) computed at ZORA-(U)M06-2X/TZ2P.

Table S5 Cartesian coordinates (in Å), ADF total energies (in kcal mol–1), and number of imaginary frequencies of the stationary points of the radical addition reactions in this study, computed at ZORA-(U)M06-2X/TZ2P.

$H_3C^•$
E = -570.9
H = -549.7
G = -563.2
N_{imag} = 0

C	-0.00000000	-0.00000000	0.00000000
H	0.53805453	0.93193777	0.00000000
H	0.53805453	-0.93193777	0.00000000
H	-1.07610904	-0.00000000	0.00000000

$MeH_2C^•$
E = -1079.9
H = -1039.5
G = -1057.3
N_{imag} = 0

C	0.86258728	0.04866934	0.00000000
H	1.41611839	-0.01991284	-0.92312338
H	1.41611839	-0.01991284	0.92312338
C	-0.62183568	0.01198744	0.00000000
H	-1.03538166	0.49812539	-0.88337562
H	-1.03538166	0.49812539	0.88337562
H	-1.00222507	-1.01708188	0.00000000

$Me_2HC^•$
E = -1589.9
H = -1530.6
G = -1551.0
N_{imag} = 0

C	0.57846132	0.21777071	0.00000000
C	-0.12907903	-0.01304947	-1.28683934
C	-0.12907903	-0.01304947	1.28683934
H	1.65485790	0.30743375	0.00000000
H	-1.10063576	0.48841463	-1.29648401
H	-0.33302025	-1.07959824	-1.45056422
H	0.44607545	0.34163085	-2.14008841
H	0.44607545	0.34163085	2.14008841
H	-0.33302025	-1.07959824	1.45056422
H	-1.10063576	0.48841463	1.29648401

$Me_3C^•$
E = -2100.4
H = -2022.2
G = -2044.2
N_{imag} = 0

C	0.00000000	0.00000000	0.23073067
C	-0.73843942	1.27901461	0.02982915
C	-0.73843942	-1.27901461	0.02982915
C	1.47687885	-0.00000000	0.02982915
H	1.94156180	-0.88502876	0.46625453
H	1.94156180	0.88502876	0.46625453
H	1.73547314	-0.00000000	-1.03924844
H	-0.20432351	-2.12395623	0.46625453
H	-0.86773656	-1.50296382	-1.03924844
H	-1.73723830	-1.23892746	0.46625453
H	-1.73723830	1.23892746	0.46625453
H	-0.86773656	1.50296382	-1.03924844
H	-0.20432351	2.12395623	0.46625453

H_2CCH_2
E = -987.6
H = -952.8
G = -968.4
N_{imag} = 0

C	-0.00000000	0.00000000	-0.66068205
C	0.00000000	0.00000000	0.66068205
H	-0.00000000	0.92184500	-1.22711338
H	0.00000000	-0.92184500	-1.22711338
H	-0.00000000	0.92184500	1.22711338
H	0.00000000	-0.92184500	1.22711338

Me_2CCMe_2
E = -3022.8
H = -2913.5
G = -2939.8
N_{imag} = 0

C	1.51745355	1.24156139	0.02807043
H	2.01719351	1.38737848	-0.93298606
H	2.30689334	1.12767839	0.77417117
H	0.96639885	2.14506524	0.26713357
H	2.00665961	-1.40397134	0.93087634
H	2.29643670	-1.14719026	-0.77670511
H	0.94810845	-2.15320500	-0.26772943
H	-2.30623494	-1.12818872	0.77540059
H	-0.96628647	-2.14527346	0.26630780
H	-2.01819307	-1.38663142	-0.93222617
H	-2.00556481	1.40369089	0.93341889
H	-2.29729869	1.14756769	-0.77393086
H	-0.94832334	2.15329552	-0.26611601
C	-0.66780447	0.00279745	0.00005966
C	0.66776514	-0.00281605	-0.00042822
C	-1.51751727	-1.24155942	0.02845504
C	-1.50699024	1.25430951	-0.02769418
C	1.50697991	-1.25426804	-0.02961100

iPr_2CCiPr_2
E = -7073.1
H = -6813.5
G = -6853.0
N_{imag} = 0

C	1.59793802	1.18838990	0.00000000
H	2.61085799	0.78523632	0.00000000
C	1.52128957	2.03095010	1.27905551
C	1.52128957	2.03095010	-1.27905551
H	0.71978239	-2.16317653	0.00000000
C	2.28193104	-1.51253191	-1.26406625
C	2.28193104	-1.51253191	1.26406625
C	-1.52128957	-2.03095010	-1.27905551
C	-1.52128957	-2.03095010	1.27905551
H	-2.61085799	-0.78523632	0.00000000
C	-2.28193104	1.51253191	-1.26406625
C	-2.28193104	1.51253191	1.26406625
H	-0.71978239	2.16317653	0.00000000
C	-0.66996844	0.02988780	0.00000000
C	0.66996844	-0.02988780	0.00000000
C	-1.59793802	-1.18838990	0.00000000
C	-1.43140406	1.34749932	0.00000000
C	1.43140406	-1.34749932	0.00000000
H	-2.77407183	2.48599368	1.26375572

H	-1.66203683	1.44285123	2.15867820
H	-3.06112898	0.75268102	1.33549981
H	0.57177046	2.55007868	1.39336361
H	2.31204179	2.78311124	1.27888684
H	1.65677610	1.39820138	2.15668089
H	2.31204179	2.78311124	-1.27888684
H	0.57177046	2.55007868	-1.39336361
H	1.65677610	1.39820138	-2.15668089
H	-1.66203683	1.44285123	-2.15867820
H	-2.77407183	2.48599368	-1.26375572
H	-3.06112898	0.75268102	-1.33549981
H	3.06112898	-0.75268102	-1.33549981
H	2.77407183	-2.48599368	-1.26375572
H	1.66203683	-1.44285123	-2.15867820
H	2.77407183	-2.48599368	1.26375572
H	1.66203683	-1.44285123	2.15867820
H	3.06112898	-0.75268102	1.33549981
H	-0.57177046	-2.55007868	-1.39336361
H	-2.31204179	-2.78311124	-1.27888684
H	-1.65677610	-1.39820138	-2.15668089
H	-2.31204179	-2.78311124	1.27888684
H	-0.57177046	-2.55007868	1.39336361
H	-1.65677610	-1.39820138	2.15668089

$H_2CCHPO(OEt)_2$

E = -4175.7
H = -4046.0
G = -4079.3
N_{imag} = 0

C	1.04132764	-1.12798828	-3.02839649
C	0.38774493	-1.50779671	-1.94101515
H	1.62212977	-0.21383725	-3.02646906
H	1.02076979	-1.71370047	-3.93820934
P	0.42867921	-0.49261013	-0.47591733
H	-0.19797841	-2.41872896	-1.91308721
O	1.28822106	0.69012448	-0.58423290
O	-1.09455275	-0.17056545	-0.15066901
O	0.79273016	-1.51888800	0.66423151
C	-1.72045867	0.93548314	-0.82481387
C	1.42412400	-1.05485923	1.87378550
H	-1.28849732	1.04946519	-1.82150160
C	-1.56479998	2.20755879	-0.02085640
H	-2.76587244	0.65629040	-0.93601160
H	1.76236574	-1.95844671	2.37382863
C	0.46597359	-0.26704360	2.74133937
H	2.28962496	-0.45313945	1.59943293
H	-0.51037093	2.46006055	0.07185939
H	-1.99265279	2.07870650	0.97245289
H	-2.08336766	3.02697764	-0.51789547
H	-0.43124442	-0.84836715	2.94614058
H	0.17633570	0.66118082	2.25033292
H	0.94976884	-0.01987609	3.68567167

RC_$H_3C^{\bullet}$+H_2CCH_2

E = -1559.6
H = -1503.0
G = -1527.2
N_{imag} = 0

C	-2.60033853	-0.71898025	-0.05193203
H	-3.52710908	-0.56537842	-0.57735628
H	-2.07422501	0.11993322	0.36895746

H	-2.21825340	-1.71529697	0.08629830
C	-0.77244588	-0.42579977	-2.81368576
C	0.54292765	-0.49686860	-2.92310856
H	-1.27800394	0.51324076	-2.63027932
H	-1.39859531	-1.30419878	-2.89951790
H	1.16699269	0.38250828	-2.83455202
H	1.04622068	-1.43735823	-3.10416625

RC_Me$_3$C$^•$+H$_2$CCH$_2$
E = -3090.1
H = -2976.1
G = -3008.1
N$_{imag}$ = 0

C	-2.31553411	-0.68819031	-0.36181545
C	-3.74573227	-0.53296794	-0.74882679
C	-1.61147197	0.46218422	0.27042585
C	-1.78053983	-2.05606740	-0.11398041
H	-0.69363037	-2.07631718	-0.21376970
H	-2.20280944	-2.78494067	-0.80743743
H	-2.01627707	-2.40576548	0.90115181
H	-0.52939421	0.36860515	0.16025689
H	-1.82020544	0.52263042	1.34798474
H	-1.92056800	1.41276728	-0.16697106
H	-3.93585561	0.44481521	-1.19462977
H	-4.41448546	-0.61975637	0.11972269
H	-4.05359255	-1.30154247	-1.45977651
C	-0.68886129	-0.39830866	-2.98182197
C	0.61748370	-0.53525621	-2.83004783
H	-1.17351683	0.56571979	-2.89723264
H	-1.32895464	-1.24505499	-3.19328633
H	1.25455748	0.31273663	-2.61586355
H	1.09884003	-1.50083839	-2.91231075

RC_H$_3$C$^•$+Me$_2$CCMe$_2$
E = -3596.1
H = -3465.1
G = -3497.6
N$_{imag}$ = 0

C	-1.42535449	-0.97949276	-1.32193838
H	-0.88387655	-0.90560382	-2.25894497
H	-1.86925090	-1.97610222	-1.25900580
H	-2.25786321	-0.27246288	-1.36328671
H	-2.09703957	0.25095819	1.09054101
H	-2.06169022	-1.49649126	1.21952479
H	-0.85062571	-0.53666802	2.05948777
H	2.25703244	0.63638528	0.82453321
H	0.98468112	0.15290319	1.93413665
H	2.18421158	-1.02278774	1.38253837
H	2.48089751	-1.21962061	-1.15285743
H	1.07848471	-1.11039072	-2.20704285
H	1.92409759	0.35122487	-1.69116433
C	0.73827783	-0.52326675	-0.12865150
C	-0.58611273	-0.69558783	-0.10369659
C	1.56807911	-0.17250642	1.07911054
C	1.58316148	-0.63744333	-1.37121355
C	-1.42640085	-0.61098076	1.14307843
C	-0.33284237	2.67486644	0.53240739
H	-1.14973059	2.54832494	-0.15678300
H	-0.52195063	2.71321239	1.59130322
H	0.66381449	2.83152987	0.15792372

RC_Me$_3$C$^•$+Me$_2$CCMe$_2$

E = -5128.3

H = -4940.3

G = -4977.1

N$_{imag}$ = 0

C	-1.01597960	-1.56689663	-1.11802347
H	-0.48282486	-1.68653767	-2.05501464
H	-1.67899642	-2.42745218	-0.99954563
H	-1.65769904	-0.68496700	-1.19494528
H	-1.74231888	-0.64850028	1.23252603
H	-1.40621098	-2.33877387	1.56604663
H	-0.36373603	-1.08049672	2.23471517
H	2.66848237	-0.40664555	1.25588021
H	1.56694046	-1.40987804	2.20323918
H	2.83160310	-2.14820640	1.22960568
H	2.54435491	-2.41415691	-1.31035264
H	1.42983333	-1.31778573	-2.12845212
H	2.78408500	-0.68365563	-1.20380955
C	1.20887104	-1.41544864	0.04531956
C	-0.12659072	-1.44457763	0.09080660
C	2.09741895	-1.33952376	1.25970324
C	2.01615228	-1.46097692	-1.22687917
C	-0.93520332	-1.37418971	1.36029299
C	-0.79312255	1.76856810	-0.08567720
C	-2.20876001	2.21971241	-0.20986469
C	-0.11547786	1.89840473	1.23534365
C	0.06060649	1.76683915	-1.30731597
H	-0.47715417	1.37870261	-2.17435582
H	0.39245931	2.78239969	-1.56639066
H	0.95620535	1.16125610	-1.15966502
H	-0.79662955	1.68629791	2.06054170
H	0.73479677	1.21664282	1.30696300
H	0.27174866	2.91545968	1.39057928
H	-2.67584015	1.83203375	-1.11694775
H	-2.80715502	1.90650199	0.64746356
H	-2.27985879	3.31585030	-0.26178692

RC_H$_3$C$^•$+iPr$_2$CCiPr$_2$

E = -7646.2

H = -7364.1

G = -7411.7

N$_{imag}$ = 0

C	2.21321500	-0.04959300	1.26436000
H	3.22339100	0.15713500	0.90986300
C	2.37303600	-1.28427000	2.16032600
C	1.81070600	1.21202400	2.03713400
H	1.52126300	-0.27334700	-2.12311200
C	2.73062000	1.30412300	-1.41382700
C	3.29471200	-1.16027600	-1.38910300
C	-0.98839300	0.37804500	-2.10771100
C	-0.30550800	-2.08941600	-2.06855000
H	-1.75421100	-1.14539700	-0.89469600
C	-1.89843300	0.21401400	1.35817900
C	-1.21779900	-2.21857100	1.47155000
H	-0.09308900	-0.55469200	2.13360000
C	0.07316900	-0.61466100	0.00630900
C	1.37430300	-0.28824700	0.00576500
C	-0.76059600	-0.87345600	-1.25184100
C	-0.73161000	-0.77687200	1.28795300
C	2.17695100	-0.11816000	-1.27585400

H	-1.75047100	-2.32125400	2.41796500
H	-0.37678500	-2.91291900	1.47450600
H	-1.90089000	-2.52249600	0.67725900
H	1.45344800	-1.57574000	2.66318900
H	3.12234600	-1.09036500	2.92971400
H	2.70879200	-2.13707100	1.56935800
H	2.49131300	1.37574900	2.87453900
H	0.79725400	1.16407200	2.43218400
H	1.86434100	2.08162300	1.38136900
H	-1.53655500	1.23908000	1.26778500
H	-2.42057500	0.11440500	2.31071600
H	-2.62907500	0.04558300	0.56592500
H	3.45141500	1.54228700	-0.63064800
H	3.23899700	1.42048200	-2.37181000
H	1.92339500	2.03589400	-1.36100200
H	3.82505200	-1.04610300	-2.33553200
H	2.88688700	-2.17076200	-1.34479100
H	4.02899000	-1.05962300	-0.58876100
H	-0.07018600	0.77048300	-2.54163800
H	-1.67625900	0.15498600	-2.92528300
H	-1.42436700	1.17197600	-1.50051900
H	-1.04359300	-2.31658700	-2.83969800
H	0.65228200	-1.94249400	-2.56332600
H	-0.21118900	-2.96348900	-1.42329400
C	-0.56231200	3.54403300	-0.09022600
H	-1.63826900	3.52185000	-0.12076600
H	-0.05547400	3.60503900	0.85718300
H	-0.00598700	3.64848800	-1.00552000

RC_$Me_3C^{\bullet}$+iPr_2CCiPr_2

E = -9177.1
H = -8838.1
G = -8890.7
N_{imag} = 0

C	2.20322234	-0.06841866	1.20263373
H	3.21284100	0.12894138	0.84120578
C	2.35967480	-1.29044914	2.11576010
C	1.80605511	1.20909949	1.95203529
H	1.50268612	-0.37041233	-2.17652040
C	2.73287985	1.21468746	-1.51911686
C	3.26161080	-1.25709900	-1.41456222
C	-0.93041913	0.52485860	-2.13081880
C	-0.48735026	-2.00011184	-2.12063644
H	-1.83196175	-0.93177785	-0.92835319
C	-1.83869616	0.38500734	1.34967557
C	-1.36418065	-2.10095761	1.40605792
H	-0.09960002	-0.55328067	2.09108953
C	0.04152078	-0.57639963	-0.03828634
C	1.35626201	-0.31266199	-0.04855045
C	-0.81943261	-0.75405905	-1.29117856
C	-0.76230420	-0.70072769	1.24793364
C	2.16028969	-0.19454758	-1.33491880
H	-1.89680023	-2.17884387	2.35483903
H	-0.58280339	-2.86170885	1.38858663
H	-2.07556808	-2.33202875	0.61206395
H	1.43337401	-1.58684029	2.60326848
H	3.09236881	-1.08075212	2.89698612
H	2.71510940	-2.14605620	1.54068706
H	2.52396541	1.42300704	2.74600921
H	0.81606908	1.15243441	2.40188446
H	1.79679680	2.05387555	1.26099832

H	-1.38714893	1.37406552	1.25979940
H	-2.35267921	0.31999374	2.30998833
H	-2.59348814	0.29075127	0.56732328
H	3.44736486	1.47280381	-0.73618319
H	3.25389400	1.29019038	-2.47452623
H	1.93411990	1.95731501	-1.50281467
H	3.79211984	-1.18161953	-2.36469783
H	2.83742500	-2.25894991	-1.33810892
H	3.99886100	-1.14312744	-0.61879870
H	-0.00442540	0.77941811	-2.64359764
H	-1.70911054	0.41417766	-2.88747668
H	-1.19135023	1.36717743	-1.48740509
H	-1.23064773	-2.13291692	-2.90871004
H	0.49122148	-1.95355374	-2.59409916
H	-0.50305210	-2.88854562	-1.48860404
C	-0.79733435	3.82023358	0.36232513
C	-2.21199256	3.80034726	-0.11177572
C	-0.56767589	4.09109214	1.81131477
C	0.25601542	4.23626938	-0.60751611
H	0.12872602	3.74293337	-1.57288317
H	0.21548195	5.31923062	-0.79507701
H	1.25737544	4.01605854	-0.23474174
H	-1.23715117	3.50192878	2.44100362
H	0.45980022	3.87772521	2.10668749
H	-0.75942358	5.14774125	2.04762256
H	-2.29485353	3.41524874	-1.12864684
H	-2.84655487	3.19680459	0.53961744
H	-2.63628810	4.81470482	-0.11807168

TS_$H_3C^{\bullet}$+H_2CCH_2

E = -1552.4

H = -1495.2

G = -1515.7

N_{imag} = 1, ν = -599.3i cm^{-1}

C	-2.05943652	-0.67360048	-0.59053283
H	-3.10012289	-0.56043281	-0.85727364
H	-1.60000722	0.14390137	-0.05736492
H	-1.72627369	-1.66669047	-0.33280703
C	-1.11268594	-0.43009789	-2.62100142
C	0.22733694	-0.54253457	-2.50294293
H	-1.57595245	0.54411263	-2.69497869
H	-1.70327646	-1.26519117	-2.97171425
H	0.84730595	0.31512797	-2.28273331
H	0.71923944	-1.50329992	-2.56109052

TS_$Me_3C^{\bullet}$+H_2CCH_2

E = -3084.4

H = -2971.0

G = -2997.5

N_{imag} = 1, ν = -533.1i cm^{-1}

C	-2.07617053	-0.67142160	-0.60059430
C	-3.51492960	-0.51235994	-0.97739538
C	-1.46389746	0.44883481	0.17734977
C	-1.63865139	-2.04588459	-0.20759936
H	-0.55016233	-2.11204167	-0.17285806
H	-2.00943170	-2.80057161	-0.90283627
H	-2.01764401	-2.30302581	0.78919075
H	-0.37745052	0.35311569	0.20688512
H	-1.82463131	0.44374739	1.21329779
H	-1.71444327	1.42048553	-0.25101032
H	-3.70851708	0.46684005	-1.41816116

H	-4.15967591	-0.60254304	-0.09404040
H	-3.83138577	-1.27923967	-1.68613605
C	-1.10818773	-0.43226545	-2.58908050
C	0.23614425	-0.54404726	-2.49283746
H	-1.56884628	0.54453848	-2.66861084
H	-1.69900095	-1.26495856	-2.94997117
H	0.85992648	0.31396266	-2.28398719
H	0.72897460	-1.50349984	-2.56674828

TS_$H_3C^{\bullet}$+Me_2CCMe_2
E = -3586.8
H = -3455.4
G = -3485.3
N_{imag} = 1, ν = -651.9i cm^{-1}

C	-1.31613688	-0.43207368	-1.27864630
H	-0.79214655	0.00218381	-2.12731398
H	-1.48147894	-1.49165176	-1.49396986
H	-2.29247559	0.04476401	-1.20787969
H	-2.36382420	0.04035769	1.11939929
H	-1.57677104	-1.49691229	1.45451447
H	-0.93841691	-0.00379231	2.14219993
H	2.29459379	0.38318726	1.29288307
H	1.04434480	-0.48747220	2.18529609
H	2.28171211	-1.36797970	1.29449520
H	2.62928794	-0.77250811	-1.07112631
H	1.18782029	-1.25144825	-1.96438025
H	1.57073786	0.45165707	-1.76310228
C	0.80358678	-0.48031577	0.02468019
C	-0.54594963	-0.27568155	0.01441561
C	1.63239453	-0.48963946	1.27271346
C	1.58665067	-0.52136973	-1.25921769
C	-1.39488466	-0.43750128	1.25655765
C	-0.57635970	1.99185943	0.02593225
H	-1.64419899	2.15917832	0.01606694
H	-0.06367096	2.20950388	0.95209145
H	-0.04481465	2.22565464	-0.88560916

TS_$Me_3C^{\bullet}$+Me_2CCMe_2
E = -5116.04
H = -4928.02
G = -4961.26
N_{imag} = 1, ν = -608.0i cm^{-1}

C	-1.23669911	-1.29010653	-1.05610156
H	-0.98386201	-0.76075505	-1.97325008
H	-1.18691566	-2.36083750	-1.26732392
H	-2.27057315	-1.05175422	-0.81299266
H	-1.95203242	-0.57910462	1.47437820
H	-1.24852829	-2.18471371	1.57682543
H	-0.41043685	-0.80861050	2.28708838
H	2.71834427	-0.26238286	0.96917736
H	1.55294429	-0.97098552	2.08732346
H	2.61842004	-2.00008331	1.13550228
H	2.16313190	-2.38064311	-1.35975000
H	1.02277208	-1.33139826	-2.19470585
H	2.51141462	-0.67263414	-1.51617054
C	1.03450345	-1.14678382	-0.02472934
C	-0.32267603	-0.93951989	0.10428431
C	2.01236359	-1.08927152	1.11105015
C	1.70038658	-1.38805340	-1.34805624
C	-1.01008860	-1.12646660	1.43998930
C	-0.50427390	1.29250765	-0.02501187

C	-1.93129945	1.59807808	-0.37788116
C	-0.09646446	1.76353291	1.34076985
C	0.48182343	1.64083211	-1.10075647
H	0.27335982	1.11423003	-2.03396771
H	0.44179033	2.71537589	-1.31944071
H	1.50206340	1.40612717	-0.79581527
H	-0.83622081	1.51119880	2.10034616
H	0.86626882	1.34522665	1.63834908
H	0.01001244	2.85633476	1.34364030
H	-2.18752389	1.26229094	-1.38232434
H	-2.63627131	1.15434942	0.32749630
H	-2.09573326	2.68402011	-0.34794287

TS_$H_3C^{\bullet}$+iPr_2CCiPr_2
E = -7630.5
H = -7348.13
G = -7389.04
N_{imag} = 1, ν = -629.0i cm^{-1}

C	2.21973699	-0.02321062	1.25907284
H	3.23949436	0.07445396	0.88746917
C	2.25858403	-1.28904764	2.12847298
C	2.00703084	1.23516211	2.11128821
H	1.49833646	-0.09384768	-2.12363691
C	2.93883763	1.27848965	-1.40866863
C	3.13671342	-1.22911716	-1.40173241
C	-0.99623783	0.39127052	-2.42317959
C	-0.31205614	-1.95723147	-1.86130939
H	-1.82248633	-0.82389751	-0.92701206
C	-2.14038290	0.11156241	1.49667955
C	-0.95463211	-2.07480847	1.37944837
H	-0.15442347	-0.24620734	2.11485666
C	-0.02674950	-0.18626833	-0.01753624
C	1.35115569	-0.12136372	0.00326828
C	-0.81223105	-0.62018626	-1.28270906
C	-0.77541657	-0.55101261	1.27920706
C	2.16939973	-0.04376046	-1.27500408
H	-1.32619164	-2.33951625	2.37026712
H	-0.01844175	-2.60472722	1.21283246
H	-1.68361942	-2.43384874	0.65085870
H	1.34913249	-1.43829844	2.70678682
H	3.08755174	-1.21809490	2.83483778
H	2.41588111	-2.17607708	1.51397873
H	2.76847799	1.27833671	2.89182508
H	1.03450590	1.25791841	2.60092596
H	2.09622627	2.13482613	1.50248541
H	-2.05045277	1.16661358	1.74094398
H	-2.64886873	-0.37774256	2.32825819
H	-2.78967335	0.02292339	0.62493068
H	3.68064441	1.40128994	-0.61881946
H	3.47048665	1.30479014	-2.36080453
H	2.26827723	2.13618321	-1.37320091
H	3.65697303	-1.19113072	-2.35994236
H	2.60535530	-2.17861611	-1.33761345
H	3.89497284	-1.21558353	-0.61769931
H	-0.06178866	0.84103751	-2.75385050
H	-1.43721561	-0.11884000	-3.28117568
H	-1.66540869	1.20055279	-2.14424723
H	-1.09233438	-2.39906590	-2.48308199
H	0.56597246	-1.82736889	-2.49194972
H	-0.05765053	-2.66986827	-1.07986821
C	-0.46711815	2.05931431	-0.02320981

H	-1.52913428	2.12651016	-0.20980568
H	-0.14929039	2.32194561	0.97426361
H	0.16957142	2.39855770	-0.82659623

TS_Me$_3$C$^•$+iPr$_2$CCiPr$_2$
E = -9142.57
H = -8802.86
G = -8847.36
N_{imag} = 1, ν = -605.7i cm^{-1}

C	2.30036118	0.10409268	1.18139247
H	3.27166509	0.31849174	0.73319612
C	2.51573713	-1.20378021	1.97039599
C	2.11540385	1.21584195	2.21833084
H	1.44784496	-0.42277075	-2.11327830
C	2.96300565	1.01508643	-1.78030658
C	3.05757708	-1.42848948	-1.22995825
C	-0.52955842	0.49968286	-2.61399418
C	-0.89550491	-1.75843684	-1.62475038
H	-1.88893175	0.02214866	-1.06967243
C	-2.26816039	-0.37253673	1.33672677
C	-0.44655214	-2.01304455	1.47652207
H	-0.31775142	0.03077503	2.09588561
C	-0.06534834	0.07720950	-0.02061518
C	1.35211035	0.02597940	-0.02876977
C	-0.86422273	-0.25420270	-1.30692893
C	-0.74356136	-0.50603625	1.24968200
C	2.14067646	-0.19591954	-1.31775748
H	-0.32851009	-2.21848004	2.54088014
H	0.45105556	-2.34606816	0.96455835
H	-1.27440682	-2.63018591	1.13177295
H	1.70705526	-1.37387187	2.67821326
H	3.43975723	-1.11599874	2.54450870
H	2.60060413	-2.07881568	1.33178324
H	2.97631464	1.20171476	2.88905495
H	1.23145993	1.06647474	2.83447850
H	2.06654079	2.20416412	1.77067047
H	-2.63432259	0.63870641	1.21272036
H	-2.59732061	-0.72905447	2.31432440
H	-2.75719869	-1.00089886	0.59128433
H	3.64755659	1.36262596	-1.00460208
H	3.56439296	0.74694860	-2.65025381
H	2.32383238	1.85000716	-2.05852697
H	3.50395857	-1.61539313	-2.20733534
H	2.49859638	-2.31913437	-0.94021708
H	3.87259215	-1.29350752	-0.51858658
H	0.02730812	-0.12496644	-3.31098835
H	-1.45233415	0.77503841	-3.12535063
H	0.04809592	1.40360744	-2.45039003
H	-1.43065280	-1.91994373	-2.56201588
H	0.11356131	-2.15847088	-1.74210551
H	-1.39795528	-2.33886997	-0.85746527
C	-0.65568071	2.24736653	0.18306916
C	-1.93417199	2.50260370	-0.58972740
C	-0.85179333	2.59486482	1.64588866
C	0.48551534	3.05035921	-0.39958161
H	0.47104834	3.06382852	-1.48784714
H	0.39599746	4.09282823	-0.06999678
H	1.45991767	2.68743946	-0.08119156
H	-1.52111371	1.92551651	2.17990195
H	0.08253362	2.64980874	2.19125182
H	-1.30272311	3.59435758	1.69440163

H	-1.84126486	2.29562734	-1.65113031
H	-2.79444781	1.95807537	-0.20622542
H	-2.17240992	3.57061301	-0.49664159

TS_$H_3C^{•}$+ $H_2CCHPO(OEt)_2$
E = -4740.2
H = -4588.0
G = -4625.4
N_{imag} = 1, ν = -481.1i cm^{-1}

C	-1.48429745	-0.72203752	-0.49237329
H	-2.54369414	-0.80941318	-0.68110300
H	-1.11363363	0.23190360	-0.14934439
H	-0.96368574	-1.61238966	-0.17638386
C	-0.72820970	-0.49169017	-2.62888345
C	0.62226021	-0.43764575	-2.57674409
H	-1.29882876	0.42736291	-2.62434111
H	-1.23672515	-1.39083811	-2.95027422
O	0.47819105	2.08102478	-1.59298941
H	1.22006895	-1.33388805	-2.69639214
P	1.41331210	1.09304270	-2.12994748
O	2.58348524	0.67372403	-1.13100980
O	2.23393461	1.60915405	-3.37452966
C	2.23509860	0.39381987	0.23262925
C	2.85385369	0.76857336	-4.35289951
C	4.07617058	0.05259524	-3.81654009
H	3.12435563	1.43995706	-5.16403843
H	2.11457762	0.06067204	-4.73201113
H	1.56649100	-0.47141913	0.25808170
H	1.69989895	1.25207499	0.63793129
C	3.50850362	0.11815899	0.99617566
H	3.80987753	-0.66381859	-3.04125980
H	4.77565346	0.76824575	-3.38866256
H	4.56905022	-0.48019165	-4.62912448
H	4.16593675	0.98449644	0.95593241
H	4.03332721	-0.73588725	0.57138809
H	3.27793057	-0.09725121	2.03850662

TS_$MeH_2C^{•}$+ $H_2CCHPO(OEt)_2$
E = -5249.9
H = -5078.6
G = -5118.4
N_{imag} = 1, ν = -432.2i cm^{-1}

C	-1.61579745	-0.64712878	-0.59504245
C	-3.08802304	-0.65802228	-0.82229766
H	-1.15697814	0.25765186	-0.22018496
H	-1.12897675	-1.57286365	-0.32111227
C	-0.76207060	-0.39765714	-2.68950760
C	0.58899368	-0.37466954	-2.62385146
H	-1.31120278	0.53478619	-2.67831388
H	-1.28929640	-1.27839315	-3.03318807
O	0.48104439	2.12882516	-1.59935850
H	1.16921838	-1.28107106	-2.75193565
P	1.40335954	1.12895504	-2.13800145
O	2.54835558	0.67002171	-1.12582713
O	2.25907274	1.65269252	-3.35664326
C	2.16692101	0.36274862	0.22234952
C	2.87199653	0.81703052	-4.34290022
C	4.06498871	0.05599943	-3.80229032
H	3.17480835	1.49783748	-5.13462561
H	2.12035806	0.13788331	-4.74934736
H	1.48266243	-0.49066814	0.21251597

H	1.63863867	1.22019614	0.63859583
C	3.41907936	0.04531385	1.00477701
H	3.76550303	-0.66927266	-3.04758786
H	4.77603328	0.74257273	-3.34668692
H	4.55738380	-0.47267403	-4.61787954
H	4.09209785	0.90056006	1.00025503
H	3.93793323	-0.80678116	0.56883456
H	3.16325593	-0.19245752	2.03634022
H	-3.41878676	0.25330945	-1.32017029
H	-3.63198168	-0.71921307	0.12615597
H	-3.39801326	-1.51269334	-1.42383880

TS_Me$_2$HC$^{\bullet}$+ H$_2$CCHPO(OEt)$_2$
E = -5761.8
H = -5571.8
G = -5613.9
N_{imag} = 1, ν = -407.5i cm^{-1}

C	-1.59468089	-0.74217532	-0.44018085
C	-3.06043529	-0.51430093	-0.58964167
H	-1.00668024	0.10511684	-0.10781106
C	-1.11929120	-2.09580031	-0.03841640
C	-0.87743930	-0.56346632	-2.58443919
C	0.47421388	-0.61325349	-2.62272600
H	-1.37340453	0.39830537	-2.59048649
H	-1.47276162	-1.43122877	-2.84124106
O	0.56359698	1.99109589	-1.90850422
H	0.99492430	-1.55491901	-2.74872241
P	1.39902385	0.87998513	-2.36110979
O	2.58710865	0.47043559	-1.37214282
O	2.21500560	1.19793171	-3.67483591
C	2.28212869	0.31352979	0.01935204
C	3.23387942	0.32439966	-4.17883034
C	4.60217261	0.90710005	-3.90195377
H	3.05126098	0.23053921	-5.24758974
H	3.13652614	-0.66536201	-3.72927086
H	1.54175930	-0.48344154	0.13420522
H	1.84468844	1.24212858	0.38571980
C	3.56087441	-0.02607479	0.74779585
H	4.76613745	0.98553282	-2.82920880
H	4.68276098	1.89873645	-4.34319154
H	5.37298102	0.26858653	-4.33281651
H	4.28953831	0.77278217	0.62208060
H	3.98790620	-0.95008313	0.36157076
H	3.36199727	-0.15159410	1.81116837
H	-3.27633595	0.46793534	-1.00734865
H	-3.56621304	-0.57594167	0.38088523
H	-3.51364297	-1.27381534	-1.23064213
H	-0.04880135	-2.21302804	-0.21073688
H	-1.64277513	-2.87640709	-0.59448816
H	-1.30405974	-2.28008866	1.02589648

TS_Me$_3$C$^{\bullet}$+ H$_2$CCHPO(OEt)$_2$
E = -6273.7
H = -6065.2
G = -6108.4
N_{imag} = 1, ν = -386.5i cm^{-1}

C	-1.85812903	-0.73048862	-0.45125157
C	-3.28488704	-0.67473038	-0.89326571
C	-1.34887932	0.44577365	0.31315087
C	-1.32979189	-2.06617158	-0.04129314
C	-0.84977312	-0.45888678	-2.45601133

C	0.50048134	-0.54364127	-2.37601954
H	-1.32014835	0.51500716	-2.51255800
H	-1.43277464	-1.30831679	-2.78852395
O	0.72545737	2.06759416	-1.67010186
H	0.99777559	-1.50306006	-2.45864673
P	1.48064854	0.92276134	-2.17618055
O	2.74006837	0.47511063	-1.29890404
O	2.19353423	1.21895194	-3.55566340
C	2.58107395	0.29646373	0.11225611
C	3.12130993	0.30610356	-4.15451669
C	4.53504551	0.82064159	-3.99468795
H	2.84201367	0.23380319	-5.20400480
H	3.01658917	-0.68410989	-3.70757518
H	1.93917047	-0.57100780	0.29008864
H	2.09089820	1.17965888	0.52376249
C	3.94918905	0.09008100	0.71875205
H	4.79576939	0.87886056	-2.93991803
H	4.62413734	1.81261747	-4.43359016
H	5.23396323	0.15190038	-4.49654010
H	4.57737343	0.95965399	0.53458382
H	4.43061711	-0.78515169	0.28571584
H	3.86229237	-0.05612294	1.79445173
H	-0.26540316	0.40975241	0.42031276
H	-1.78455284	0.45350442	1.32033910
H	-1.59727747	1.38912220	-0.17144069
H	-0.23977363	-2.05435463	0.01166445
H	-1.63658678	-2.85383863	-0.73080766
H	-1.70373213	-2.33629920	0.95372389
H	-3.51931374	0.27896652	-1.36743335
H	-3.95343199	-0.77898562	-0.02986361
H	-3.52750218	-1.48058619	-1.58722580

$H_3CH_2CH_2C^{•}$
E = -1586.5
H = -1527.0
G = -1547.2
N_{imag} = 0

C	-1.84688830	-0.63568139	-0.93070754
H	-2.93414268	-0.56279689	-0.90845412
H	-1.43815099	0.12875001	-0.27073287
H	-1.55984513	-1.60860557	-0.53321853
C	-1.31192157	-0.45677288	-2.36185677
C	0.17051114	-0.55000648	-2.42704194
H	-1.64134287	0.51266046	-2.73998251
H	-1.76180271	-1.21722866	-3.00230723
H	0.78536859	0.30849882	-2.20214916
H	0.65893877	-1.50956693	-2.50539279

$Me_3CH_2CH_2C^{•}$
E = -3111.2
H = -2996.2
G = -3021.9
N_{imag} = 0

C	-1.94358328	-0.64124219	-0.86278435
C	-3.46054902	-0.51417778	-0.99054803
C	-1.41792957	0.44372562	0.07588063
C	-1.59235698	-2.01941074	-0.30428242
H	-0.51446474	-2.12986714	-0.18055431
H	-1.94187706	-2.81051554	-0.97057380
H	-2.06063951	-2.16795900	0.67003238
H	-0.33795256	0.36241135	0.20396775

H	-1.88184586	0.35613842	1.05962309
H	-1.64092024	1.43853199	-0.31474620
H	-3.73832202	0.46293468	-1.38960470
H	-3.94131216	-0.63008465	-0.01781520
H	-3.86162402	-1.27882792	-1.65809099
C	-1.32192145	-0.46707925	-2.27569164
C	0.15965223	-0.56196913	-2.33316915
H	-1.64457703	0.50601505	-2.65675607
H	-1.76608944	-1.22791986	-2.92387001
H	0.78048994	0.30804527	-2.18586759
H	0.65312275	-1.51279472	-2.46154550

$H_3CMe_2CMe_2C^•$
E = -3621.8
H = -3487.7
G = -3515.6
N_{imag} = 0

C	-1.31885329	-0.27675763	-1.29321184
H	-0.82752194	0.16967112	-2.15775025
H	-1.35038398	-1.35780785	-1.43799083
H	-2.34547512	0.09020805	-1.26649152
H	-2.44646874	-0.12247884	1.13499380
H	-1.45417566	-1.58659615	1.11603676
H	-1.00906462	-0.22156371	2.14374400
H	2.21660588	0.74300088	1.13514325
H	1.09268525	-0.14372476	2.15898274
H	2.41511635	-0.98591116	1.35221967
H	2.51461903	-1.14318824	-1.06330262
H	0.99712333	-1.30426592	-1.94891701
H	1.74689919	0.27337589	-1.76697397
C	0.81155588	-0.45719664	0.03366741
C	-0.60384670	0.08130285	0.01244905
C	1.66446498	-0.20221263	1.23489058
C	1.54740615	-0.67626038	-1.24869213
C	-1.42255881	-0.49796004	1.17498902
C	-0.56268086	1.61500570	0.14697530
H	-1.57206699	2.02924815	0.11073356
H	-0.10899291	1.91326962	1.09273398
H	0.01561366	2.06084161	-0.66422891

$Me_3CMe_2CMe_2C^•$
E = -5140.3
H = -4950.2
G = -4982.2
N_{imag} = 0

C	-1.23318308	-1.17229402	-1.07474986
H	-1.01347765	-0.72814748	-2.04462485
H	-1.03573397	-2.24260492	-1.14206485
H	-2.29761499	-1.04111149	-0.88631860
H	-2.07487461	-0.72545311	1.46061845
H	-1.06043712	-2.16312021	1.36258465
H	-0.51270690	-0.75422450	2.26375803
H	2.67028628	-0.13746562	1.02575502
H	1.50544722	-0.90784429	2.09940974
H	2.61868793	-1.88175239	1.14215290
H	2.19196890	-2.36113984	-1.25418971
H	0.99632124	-1.43939778	-2.16142106
H	2.46203933	-0.66222440	-1.57295760
C	1.02753276	-1.05704571	-0.01943456
C	-0.40511749	-0.55914570	0.06963391
C	1.98435645	-0.99069958	1.12760783

C	1.68338147	-1.39390327	-1.32136234
C	-1.04500526	-1.07246325	1.37174339
C	-0.47516062	1.02313295	-0.00271477
C	-1.89821913	1.50834643	-0.30886112
C	-0.04095729	1.66687874	1.31922338
C	0.45368062	1.54571972	-1.10425135
H	0.27434691	1.05237861	-2.06029876
H	0.29331891	2.61551041	-1.24746243
H	1.50174763	1.39862397	-0.84034126
H	-0.75842104	1.47465781	2.11668981
H	0.93688172	1.31366803	1.64358490
H	0.02439810	2.74867751	1.19116973
H	-2.20781272	1.24998161	-1.32085065
H	-2.62915994	1.09711658	0.38807576
H	-1.93651375	2.59534511	-0.22010382

$H_3CiPr_2CiPr_2C^{\bullet}$
E = -7664.0
H = -7379.0
G = -7418.5
N_{imag} = 0

C	2.23244227	-0.14573164	1.27427898
H	3.26009058	-0.14249974	0.90806976
C	2.17259544	-1.37663353	2.19198740
C	2.11678218	1.14435065	2.10631128
H	1.49134336	0.07938194	-2.09648951
C	2.98246385	1.34026173	-1.26629995
C	3.14250144	-1.14203531	-1.53420486
C	-1.10508225	0.25005184	-2.50097164
C	-0.21889086	-1.95315900	-1.75570302
H	-1.84031693	-0.88919703	-0.90748129
C	-2.22224115	0.10802208	1.50955407
C	-0.92102382	-2.00406003	1.35465253
H	-0.21354982	-0.14177971	2.09965397
C	-0.13268344	0.04506346	-0.03601803
C	1.38058435	-0.18657942	0.01178453
C	-0.84444390	-0.63591960	-1.27433057
C	-0.82704331	-0.47861652	1.26643963
C	2.18192566	0.02469756	-1.25930506
H	-1.23960098	-2.29430171	2.35688100
H	0.02891266	-2.49219192	1.15387092
H	-1.65862981	-2.39751615	0.65336759
H	1.24951313	-1.43858589	2.76456669
H	2.99724605	-1.33062960	2.90533019
H	2.27863789	-2.29626425	1.61605319
H	2.88919365	1.14955211	2.87728996
H	1.15492027	1.23465891	2.60962833
H	2.25567040	2.02864644	1.48473534
H	-2.19369447	1.17698902	1.71041590
H	-2.67323521	-0.37624621	2.37614046
H	-2.89132604	-0.05755414	0.66313827
H	3.75425790	1.34152729	-0.49550785
H	3.48290413	1.46609527	-2.22747174
H	2.34436447	2.20729369	-1.10142666
H	3.64662957	-1.00356998	-2.49187781
H	2.61659331	-2.09521253	-1.55618743
H	3.91455430	-1.20454097	-0.76589711
H	-0.19019469	0.62699930	-2.95764060
H	-1.62045150	-0.34365216	-3.25754400
H	-1.73753066	1.10510675	-2.27276567
H	-0.97817222	-2.55585525	-2.25617451

H	0.57427162	-1.77443416	-2.48094386
H	0.20073714	-2.54510927	-0.94718047
C	-0.35336166	1.57482665	-0.11372278
H	-1.40640709	1.82360592	-0.23732967
H	0.00129522	2.06143238	0.79372958
H	0.18332762	1.99929143	-0.96053574

$Me_3CiPr_2CiPr_2C^•$
E = -9158.5
H = -8816.6
G = -8860.0
N_{imag} = 0

C	2.31919956	0.17002422	1.19495829
H	3.28254948	0.35647281	0.71539814
C	2.53115309	-1.13530885	1.99619819
C	2.21315643	1.27395045	2.25621925
H	1.38774759	-0.49703737	-2.05261961
C	2.96111521	0.88975580	-1.82677382
C	2.98595787	-1.51808002	-1.16085164
C	-0.53333108	0.50512629	-2.67149266
C	-0.83291601	-1.71796804	-1.59070962
H	-1.92930558	0.01281244	-1.16149381
C	-2.32168512	-0.49585881	1.31830310
C	-0.36525377	-1.98237134	1.33994266
H	-0.39850539	0.00179641	2.09342968
C	-0.18346915	0.31124267	-0.00865153
C	1.35124446	0.08966621	0.00035358
C	-0.87834239	-0.20278425	-1.33391789
C	-0.78988357	-0.50023637	1.20836878
C	2.10249264	-0.26424630	-1.27934271
H	-0.19542965	-2.22503446	2.38847669
H	0.54044280	-2.22227395	0.79208690
H	-1.14868863	-2.65216610	0.99408623
H	1.72253046	-1.29012378	2.70793025
H	3.45616403	-1.04489644	2.56841928
H	2.60946454	-2.01981593	1.37225442
H	3.14401940	1.27771210	2.82551575
H	1.41554449	1.07149109	2.96947148
H	2.07775054	2.27062087	1.84761472
H	-2.77319641	0.48958211	1.31431293
H	-2.60849316	-0.98413838	2.25132686
H	-2.77120689	-1.07258671	0.50889930
H	3.69651562	1.22993873	-1.09580149
H	3.50832173	0.55846794	-2.71059520
H	2.35335943	1.74578074	-2.11028093
H	3.40222936	-1.75925705	-2.13969367
H	2.41542747	-2.38258804	-0.81892256
H	3.82265458	-1.37283938	-0.47709047
H	0.02633980	-0.14974929	-3.33758336
H	-1.45172848	0.76408142	-3.19954801
H	0.04779080	1.41243922	-2.55832605
H	-1.21948240	-1.91152457	-2.59224890
H	0.17535034	-2.12891033	-1.54232950
H	-1.45404087	-2.27787457	-0.90072288
C	-0.57749695	1.90371035	0.16834789
C	-1.87865144	2.32055316	-0.55580783
C	-0.80771042	2.34932256	1.63002011
C	0.51488695	2.83575114	-0.39127877
H	0.55120346	2.82910342	-1.47727298
H	0.29331241	3.86216624	-0.09365275
H	1.50891564	2.58903789	-0.02999148

H	-1.66069455	1.86382266	2.09488754
H	0.04953182	2.20218642	2.26953066
H	-1.01883622	3.41935799	1.62419551
H	-1.81697973	2.23193290	-1.63583534
H	-2.75293402	1.76825893	-0.21856492
H	-2.06119310	3.37353670	-0.33489778

Table S6 Cartesian coordinates (in Å), ADF total energies (in kcal mol[–1]), and number of imaginary frequencies of the stationary points of the radical addition reactions in this study, computed at ZORA-(U)OLYP-D3(BJ)/TZ2P.

$H_3C^•$
E = -410.5
H = -389.6
G = -403.1
N_{imag} = 0

C	0.00000000	0.00000000	0.00000000
H	0.54131400	0.93758400	0.00000000
H	0.54131400	-0.93758400	0.00000000
H	-1.08262900	0.00000000	0.00000000

$Me_3C^•$
E = -1548.2
H = -1471.8
G = -1493.6
N_{imag} = 0

C	-0.00000000	-0.00000000	0.22037800
C	-0.73653200	1.27571000	0.03233700
C	-0.73653200	-1.27571000	0.03233700
C	1.47306300	-0.00000000	0.03233700
H	1.93831500	-0.89023400	0.46816400
H	1.93831500	0.89023400	0.46816400
H	1.74033800	-0.00000000	-1.04212400
H	-0.19819200	-2.12374700	0.46816400
H	-0.87016900	-1.50717700	-1.04212400
H	-1.74012200	-1.23351300	0.46816400
H	-1.74012200	1.23351300	0.46816400
H	-0.87016900	1.50717700	-1.04212400
H	-0.19819200	2.12374700	0.46816400

H_2CCH_2
E = -724.6
H = -690.6
G = -706.3
N_{imag} = 0

C	0.00000000	0.00000000	-0.66542300
C	-0.00000000	0.00000000	0.66542300
H	-0.00000000	0.92627700	-1.23446200
H	0.00000000	-0.92627700	-1.23446200
H	-0.00000000	0.92627700	1.23446200
H	0.00000000	-0.92627700	1.23446200

Me_2CCMe_2
E = -2236.1
H = -2129.1
G = -2155.8
N_{imag} = 0

C	1.50300084	1.22915697	0.16696686
H	0.92226632	2.13676699	0.31650917
H	2.11918039	1.35832842	-0.73237556
H	2.20196499	1.13603210	1.00683610
H	1.94896274	-1.39043721	1.10910105
H	2.34965634	-1.16798992	-0.58533142
H	0.96518098	-2.16905668	-0.14176332
H	-2.34925885	-1.16866763	0.58553951
H	-0.96460845	-2.16932304	0.14159278
H	-1.94878565	-1.39074935	-1.10899145

H	-2.20231605	1.13553362	-1.00656003
H	-0.92279737	2.13655266	-0.31630892
H	-2.11943833	1.35779985	0.73265243
C	-0.66980656	-0.01634710	-0.06194939
C	0.66981120	-0.01618584	0.06206510
C	-1.50807156	-1.26169933	-0.11184987
C	-1.50330555	1.22880120	-0.16674690
C	1.50838100	-1.26133854	0.11190679

iPr_2CCiPr_2
E = -5238.9
H = -4983.6
G = -5023.5
N_{imag} = 0

C	1.59240375	1.18749873	0.00000000
H	2.60534356	0.78099165	0.00000000
C	1.51623076	2.03215962	1.27431837
C	1.51623076	2.03215962	-1.27431837
H	0.71195481	-2.14859747	0.00000000
C	2.27930538	-1.50956687	-1.26105480
C	2.27930538	-1.50956687	1.26105480
C	-1.51623076	-2.03215962	-1.27431837
C	-1.51623076	-2.03215962	1.27431837
H	-2.60534356	-0.78099165	0.00000000
C	-2.27930538	1.50956687	-1.26105480
C	-2.27930538	1.50956687	1.26105480
H	-0.71195481	2.14859747	0.00000000
C	-0.67217779	0.02637700	0.00000000
C	0.67217779	-0.02637700	0.00000000
C	-1.59240375	-1.18749873	0.00000000
C	-1.42994881	1.33732770	0.00000000
C	1.42994881	-1.33732770	0.00000000
H	-2.76563901	2.48967702	1.25495763
H	-1.65847764	1.43621612	2.15654924
H	-3.06297139	0.75181166	1.32874859
H	0.57037799	2.56397760	1.37187550
H	2.32034110	2.77527905	1.27657564
H	1.63312833	1.39796072	2.15542964
H	2.32034110	2.77527905	-1.27657564
H	0.57037799	2.56397760	-1.37187550
H	1.63312833	1.39796072	-2.15542964
H	-1.65847764	1.43621612	-2.15654924
H	-2.76563901	2.48967702	-1.25495763
H	-3.06297139	0.75181166	-1.32874859
H	3.06297139	-0.75181166	-1.32874859
H	2.76563901	-2.48967702	-1.25495763
H	1.65847764	-1.43621612	-2.15654924
H	2.76563901	-2.48967702	1.25495763
H	1.65847764	-1.43621612	2.15654924
H	3.06297139	-0.75181166	1.32874859
H	-0.57037799	-2.56397760	-1.37187550
H	-2.32034110	-2.77527905	-1.27657564
H	-1.63312833	-1.39796072	-2.15542964
H	-2.32034110	-2.77527905	1.27657564
H	-0.57037799	-2.56397760	1.37187550
H	-1.63312833	-1.39796072	2.15542964

RC_$Me_3C^{\bullet}$+H_2CCH_2
E = -2274.9
H = -2163.3
G = -2195.4

N_{imag} = 0

C	-2.26306304	-0.69728453	-0.32420622
C	-3.63539452	-0.52411972	-0.85823613
C	-1.60218195	0.44735648	0.34783925
C	-1.76902778	-2.06539635	-0.03666194
H	-0.67496558	-2.10037163	-0.02568634
H	-2.13484427	-2.79109107	-0.76975185
H	-2.10820014	-2.41714703	0.95553672
H	-0.51324914	0.33522658	0.34849500
H	-1.91368972	0.52927704	1.40589569
H	-1.85571194	1.39924650	-0.12919513
H	-3.76640864	0.45702389	-1.32654493
H	-4.39543609	-0.59320275	-0.05726396
H	-3.88511984	-1.29867282	-1.59084576
C	-0.78627509	-0.39543547	-2.95640850
C	0.53926660	-0.51653596	-2.92425934
H	-1.27453034	0.56462744	-2.82618988
H	-1.43326609	-1.25069803	-3.12065033
H	1.18538679	0.34145485	-2.75740666
H	1.02616290	-1.47980478	-3.05268792

RC_$Me_3C^{\bullet}$+Me_2CCMe_2
E = -3789.0
H = -3604.0
G = -3644.8
N_{imag} = 0

C	-1.00392395	-1.62682512	-1.13137501
H	-0.44769745	-1.73283600	-2.05948355
H	-1.66760452	-2.49497341	-1.03578094
H	-1.64998479	-0.74704527	-1.21490565
H	-1.75731566	-0.71667197	1.23182742
H	-1.46141454	-2.42649917	1.51387803
H	-0.39681905	-1.21162273	2.23766276
H	2.64937470	-0.46461867	1.29560989
H	1.50984629	-1.44595651	2.22852548
H	2.79215248	-2.21254355	1.28773793
H	2.61197131	-2.41415492	-1.24598354
H	1.43958866	-1.40134233	-2.10142906
H	2.74921014	-0.66680811	-1.17434169
C	1.20214371	-1.46709769	0.06736538
C	-0.14168030	-1.51176838	0.09114779
C	2.06350293	-1.39248354	1.29434549
C	2.02390701	-1.48924437	-1.18891987
C	-0.96470173	-1.46252240	1.34541944
C	-0.76216051	1.87070637	-0.10201985
C	-2.20154598	2.22458983	-0.16272422
C	-0.04457854	2.01130305	1.18843632
C	0.03248334	1.88379099	-1.35439277
H	-0.54092366	1.49827655	-2.20272574
H	0.34354984	2.91117693	-1.62292247
H	0.94436627	1.29119685	-1.25317185
H	-0.67112168	1.71539636	2.03491336
H	0.86752488	1.41034518	1.20390832
H	0.25782086	3.06036932	1.36846134
H	-2.68625222	1.80373229	-1.04986580
H	-2.74093433	1.88367162	0.72725892
H	-2.34878343	3.32045877	-0.21645590

RC_$Me_3C^{\bullet}$+iPr_2CCiPr_2
E = -6791.8
H = -6458.5

G = -6512.5
N N_{imag} = 0

C	2.38115092	-0.30979367	1.17971973
H	3.38639154	-0.10031848	0.80946445
C	2.55528112	-1.52088620	2.09926908
C	1.97583508	0.95692822	1.93666247
H	1.67304376	-0.65192211	-2.17984064
C	2.89019566	0.95281418	-1.55186515
C	3.45287244	-1.50299325	-1.42707254
C	-0.79538287	0.08587056	-2.18201302
C	-0.20330824	-2.39434379	-2.08927089
H	-1.60876455	-1.37477930	-0.92453582
C	-1.69721842	0.01365179	1.29351953
C	-1.08710141	-2.43066789	1.44482605
H	0.09523307	-0.79286733	2.06069454
C	0.23380325	-0.89076309	-0.05456326
C	1.54234813	-0.57938011	-0.06282924
C	-0.60813758	-1.14901835	-1.29723331
C	-0.56257413	-1.01008354	1.22766166
C	2.33731077	-0.45897380	-1.34598803
H	-1.61389228	-2.49456302	2.40180533
H	-0.26477352	-3.14941192	1.45266749
H	-1.78819996	-2.72602908	0.66096031
H	1.63371186	-1.80146663	2.60789246
H	3.30678659	-1.30110957	2.86470876
H	2.89401023	-2.38549584	1.52472570
H	2.71116042	1.17739447	2.71746294
H	0.99915470	0.87055995	2.41041952
H	1.93497982	1.80547358	1.25319821
H	-1.31502971	1.02306472	1.14393412
H	-2.18938840	-0.03555411	2.26969179
H	-2.45724032	-0.17447507	0.53201617
H	3.60497595	1.22619910	-0.77245104
H	3.40949045	1.01509534	-2.51299378
H	2.08370217	1.68692187	-1.54492976
H	3.97057764	-1.42638937	-2.38802233
H	3.04415022	-2.51133307	-1.33255682
H	4.19593562	-1.36313781	-0.63877225
H	0.12194764	0.38083335	-2.69081362
H	-1.55369487	-0.11361194	-2.94641330
H	-1.12678810	0.93057630	-1.57847102
H	-0.95571628	-2.60781941	-2.85563077
H	0.75867119	-2.28438426	-2.58868804
H	-0.13578822	-3.25871456	-1.42535323
C	-1.08434774	3.57442359	0.32401745
C	-2.47493554	3.48698575	-0.18786328
C	-0.87975407	3.86529315	1.76530916
C	0.00240347	3.91242365	-0.62854971
H	-0.11444874	3.38666633	-1.58050021
H	0.00558210	4.99400882	-0.86317706
H	0.98841856	3.67911940	-0.21807667
H	-1.59936442	3.32988399	2.39226175
H	0.13095134	3.60532456	2.09209903
H	-1.01554406	4.94251556	1.97849956
H	-2.51615503	3.01760910	-1.17508410
H	-3.12214495	2.92654651	0.49383885
H	-2.92507385	4.49192569	-0.29607736

TS_$H_3C^{\bullet}$+H_2CCH_2
E = -1133.3
H = -1077.6

G = -1098.8n

N_{imag} = 1, ν = -263.4i cm^{-1}

C	-2.13103562	-0.68416874	-0.48996647
H	-3.15241725	-0.56188995	-0.82892445
H	-1.63838622	0.15133666	-0.01108656
H	-1.76513496	-1.68241613	-0.29068196
C	-1.07094776	-0.42237705	-2.68795658
C	0.26287846	-0.53814578	-2.54818881
H	-1.55181970	0.54975289	-2.70346320
H	-1.68051214	-1.26998379	-2.98208271
H	0.88636519	0.32301265	-2.32520130
H	0.75713717	-1.50382610	-2.60488748

TS_$Me_3C^{\bullet}$+H_2CCH_2

E = -2274.0

H = -2162.7

G = -2189.9

N_{imag} = 1, ν = -208.0i cm^{-1}

C	-2.11992731	-0.67917705	-0.52904525
C	-3.53410673	-0.51467950	-0.95537681
C	-1.48657617	0.44807058	0.20383501
C	-1.66040558	-2.04933115	-0.18206304
H	-0.56884249	-2.10537742	-0.16506383
H	-2.03900965	-2.79401752	-0.88800087
H	-2.02032384	-2.33830018	0.82015174
H	-0.39806285	0.34816639	0.21430170
H	-1.82506015	0.46856290	1.25383397
H	-1.74607016	1.41449590	-0.23767154
H	-3.70962817	0.46809109	-1.40292046
H	-4.21578445	-0.59954880	-0.09122030
H	-3.83249628	-1.28548008	-1.67213199
C	-1.05514157	-0.42342492	-2.66316648
C	0.28379608	-0.53874701	-2.56033357
H	-1.53238930	0.54967690	-2.67938262
H	-1.66681235	-1.26616312	-2.96417563
H	0.91201862	0.32050321	-2.34531626
H	0.77684188	-1.50365465	-2.63139708

TS_$H_3C^{\bullet}$+Me_2CCMe_2

E = -2645.4

H = -2516.5

G = -2547.4

N_{imag} = 1, ν = -312.9i cm^{-1}

C	2.21554036	-0.05994599	-1.30744711
H	1.72804456	0.42365573	-2.15236868
H	2.35759464	-1.11933126	-1.55732147
H	3.20632867	0.38285220	-1.19222248
H	3.17628501	0.53585699	1.12814379
H	2.57714873	-1.10955348	1.32689936
H	1.74885655	0.23383443	2.11913980
H	-1.51617803	0.82541110	1.10722058
H	-0.19850423	0.21233177	2.11709619
H	-1.33328655	-0.90414308	1.34735571
H	-1.66461954	-0.63330438	-1.13223025
H	-0.19533165	-0.56740254	-2.11486116
H	-1.00366155	0.92380576	-1.61564071
C	0.06956821	-0.04210065	-0.02537016
C	1.42756244	0.07691011	-0.03027105
C	-0.76874319	0.02704165	1.20954965
C	-0.72813435	-0.09096309	-1.29238377
C	2.26693440	-0.06279587	1.20969110

C	1.46148757	2.50769489	-0.02346544
H	2.53436513	2.60586136	-0.11748346
H	1.01518370	2.66234914	0.94940622
H	0.85330451	2.68236364	-0.90056607

TS_Me$_3$C$^•$+Me$_2$CCMe$_2$
E = -3783.4
H = -3598.6
G = -3632.8
N_{imag} = 1, ν = -334.5i cm^{-1}

C	2.33519859	-0.11549945	-1.17846226
H	2.04699335	0.39735482	-2.09469187
H	2.30980944	-1.19303371	-1.37976245
H	3.36711112	0.15304968	-0.95602431
H	3.08362216	0.58102433	1.32705264
H	2.36499109	-1.02014368	1.46243873
H	1.54502953	0.37658478	2.16125309
H	-1.63057426	0.86534091	0.87408478
H	-0.39549111	0.25909026	1.98639853
H	-1.43163148	-0.86175633	1.09669759
H	-1.01305529	-1.21505082	-1.47064762
H	0.05464313	-0.07498005	-2.29297035
H	-1.45898696	0.47938072	-1.56307670
C	0.07593163	0.03960153	-0.12507416
C	1.43817043	0.21588281	-0.01053844
C	-0.87401253	0.08304216	1.02634512
C	-0.60304538	-0.19471664	-1.43576515
C	2.13532249	0.04276121	1.31252488
C	1.64107415	2.54558813	-0.14097140
C	3.07389788	2.81786973	-0.45336863
C	1.19811228	2.98879582	1.21313347
C	0.68503131	2.86408240	-1.24032904
H	0.95465309	2.36674938	-2.17420104
H	0.68554149	3.94860484	-1.43998572
H	-0.33380739	2.57955661	-0.97717428
H	1.89957528	2.68825438	1.99323697
H	0.20887839	2.60118585	1.45891879
H	1.13414176	4.08994828	1.24404686
H	3.35721118	2.44783906	-1.43999081
H	3.74817226	2.38803138	0.29171662
H	3.25601202	3.90710319	-0.45212903

TS_H$_3$C$^•$+iPr$_2$CCiPr$_2$
E = -5641.0
H = -5362.9
G = -5404.7
N_{imag} = 1, ν = -314.2i cm^{-1}

C	2.21252172	-0.01584963	1.25394365
H	3.22933669	0.10322500	0.87735502
C	2.26970596	-1.29317630	2.09931237
C	1.98694895	1.21890374	2.12924478
H	1.48495385	-0.09854619	-2.10958813
C	2.92497163	1.28228858	-1.41317732
C	3.13320231	-1.21737746	-1.38838738
C	-0.99628673	0.37184267	-2.40329992
C	-0.30602506	-1.97266332	-1.86002294
H	-1.81309466	-0.84950379	-0.91355536
C	-2.12510271	0.11092301	1.47761668
C	-0.96644453	-2.08555849	1.38270975
H	-0.14618553	-0.26444468	2.10319222
C	-0.02235762	-0.22188211	-0.01779075

C	1.34826050	-0.11396229	0.00616598
C	-0.80469465	-0.64232110	-1.27445184
C	-0.77173859	-0.56710543	1.27040545
C	2.16160305	-0.03810350	-1.26572728
H	-1.34539493	-2.33449122	2.37785746
H	-0.03274767	-2.62161410	1.22367853
H	-1.69586110	-2.44087925	0.65244014
H	1.36396296	-1.45080684	2.68192029
H	3.10842260	-1.22525965	2.79949909
H	2.42354258	-2.16899196	1.46722139
H	2.74631836	1.24641122	2.91691039
H	1.00937097	1.21921941	2.61096535
H	2.07300085	2.13121811	1.53890556
H	-2.01956833	1.17456466	1.67821009
H	-2.62933118	-0.34750188	2.33307456
H	-2.77989443	-0.00782692	0.61188234
H	3.66555668	1.41164886	-0.62115027
H	3.45845967	1.29456576	-2.36812977
H	2.25010218	2.13748106	-1.38596611
H	3.64276203	-1.18128775	-2.35583866
H	2.60661978	-2.16893016	-1.30891605
H	3.89838704	-1.18624166	-0.60998667
H	-0.05616510	0.78424036	-2.76765886
H	-1.48840665	-0.12595606	-3.24443846
H	-1.62325965	1.20452948	-2.09310996
H	-1.08795456	-2.40713911	-2.49037766
H	0.57694337	-1.83547410	-2.48236249
H	-0.05815639	-2.68756739	-1.07854717
C	-0.45544210	2.15710650	-0.02817405
H	-1.51786044	2.21146935	-0.21459784
H	-0.11987598	2.37161605	0.97334533
H	0.19840860	2.43220893	-0.84029732

TS_Me$_3$C$^•$+iPr$_2$CCiPr$_2$
E = -6762.2
H = -6427.6
G = -6473.1
N_{imag} = 1, ν = -306.4i cm^{-1}

C	2.30369836	0.11165722	1.18001934
H	3.26753893	0.33942694	0.71959318
C	2.55186730	-1.19754002	1.95344961
C	2.11809800	1.20704203	2.22722920
H	1.41906205	-0.36947100	-2.09243224
C	2.98342755	1.00384866	-1.75352612
C	3.00826516	-1.44081342	-1.24687404
C	-0.52721708	0.49729424	-2.60501061
C	-0.87642200	-1.75313584	-1.61769946
H	-1.87593470	0.01747850	-1.05992616
C	-2.27328066	-0.37158305	1.31291855
C	-0.42875204	-1.97695842	1.49509104
H	-0.34432008	0.07240537	2.08497472
C	-0.06689737	0.08704267	-0.01637321
C	1.35418492	0.03549201	-0.01447727
C	-0.85108171	-0.25282760	-1.29955859
C	-0.75182892	-0.48510795	1.24539385
C	2.12795873	-0.18406033	-1.30089884
H	-0.32173171	-2.16692975	2.56568779
H	0.48243390	-2.28683382	0.99688778
H	-1.23928576	-2.61412186	1.14065174
H	1.76251305	-1.37842842	2.67986349
H	3.49367592	-1.10110622	2.50259276

H	2.62768330	-2.06468595	1.30342914
H	2.99236559	1.20415407	2.88539992
H	1.24612064	1.02747045	2.85406435
H	2.03663617	2.19680520	1.78663728
H	-2.64465170	0.63521432	1.16932002
H	-2.60820200	-0.71841274	2.29496087
H	-2.74440444	-1.01615446	0.56901203
H	3.71570219	1.28856921	-0.99393393
H	3.53686870	0.73400834	-2.65794020
H	2.37097546	1.87454841	-1.97536322
H	3.43287364	-1.62360163	-2.23832210
H	2.42846704	-2.31823638	-0.95653700
H	3.83750520	-1.33081208	-0.54656269
H	0.05853076	-0.11702877	-3.29085841
H	-1.45734639	0.73593880	-3.12729646
H	0.01709703	1.41893286	-2.44012533
H	-1.44046939	-1.91476863	-2.54144791
H	0.13297586	-2.13839890	-1.76682334
H	-1.34797527	-2.33630720	-0.83291319
C	-0.67460250	2.25409649	0.17889540
C	-1.93968219	2.50082254	-0.60053065
C	-0.87895957	2.58035326	1.63502366
C	0.48014319	3.02933388	-0.39212759
H	0.48357151	3.02094677	-1.48074069
H	0.40095927	4.08141144	-0.08071742
H	1.43857330	2.65230455	-0.04859854
H	-1.64347736	1.97518660	2.11582372
H	0.03306313	2.51117544	2.21461475
H	-1.21775653	3.62614454	1.69834645
H	-1.82544644	2.32130541	-1.66494449
H	-2.79146724	1.92624597	-0.23995582
H	-2.20546466	3.56367629	-0.48268123

$H_3CH_2CH_2C^{\bullet}$
E = -1163.8
H = -1105.5
G = -1126.2
N_{imag} = 0

C	-1.85028543	-0.63612477	-0.92639558
H	-2.94267935	-0.55987466	-0.92071747
H	-1.44520599	0.13200833	-0.26190874
H	-1.56826318	-1.61369139	-0.52582838
C	-1.30401497	-0.45831602	-2.35659404
C	0.17312277	-0.55032517	-2.42640727
H	-1.63859345	0.51678914	-2.73151752
H	-1.75982211	-1.22426554	-2.99562868
H	0.79172358	0.31603579	-2.21607613
H	0.66474239	-1.51298523	-2.52076965

$Me_3CH_2CH_2C^{\bullet}$
E = -2294.4
H = -2181.3
G = -2207.1
N_{imag} = 0

C	-1.94441028	-0.64163223	-0.85962319
C	-3.45879992	-0.51397218	-0.99274421
C	-1.41843960	0.44371653	0.07496744
C	-1.59263707	-2.01882772	-0.30493586
H	-0.51163757	-2.12748574	-0.19423935
H	-1.95113249	-2.80810532	-0.97221434
H	-2.05554048	-2.16433579	0.67503406

H	-0.33527062	0.36476739	0.18961728
H	-1.87712068	0.35047486	1.06325661
H	-1.65124828	1.43824201	-0.31676713
H	-3.73285038	0.46624142	-1.39319538
H	-3.94007337	-0.63065929	-0.01750667
H	-3.85641139	-1.28114420	-1.66329741
C	-1.31694695	-0.46754734	-2.27528819
C	0.15630972	-0.56119193	-2.33883880
H	-1.64663448	0.50926271	-2.64816026
H	-1.76907662	-1.23322313	-2.91658446
H	0.77877438	0.31035096	-2.17324925
H	0.65044604	-1.51897654	-2.45262701

$H_3CMe_2CMe_2C^{\bullet}$
E = -2675.3
H = -2543.6
G = -2571.8n
N_{imag} = 0

C	-1.31962052	-0.27289708	-1.29344402
H	-0.83022101	0.17933938	-2.15760161
H	-1.34925604	-1.35550753	-1.43963149
H	-2.34810435	0.09430180	-1.25685333
H	-2.44024768	-0.12897324	1.13225300
H	-1.43821158	-1.59329314	1.10895366
H	-0.99480856	-0.22368809	2.14009549
H	2.24403516	0.73664694	1.13344338
H	1.08344857	-0.11764240	2.15148908
H	2.39814658	-0.99955070	1.35623197
H	2.50079987	-1.18723409	-1.04848606
H	0.97836621	-1.28235094	-1.95236692
H	1.79655507	0.26347251	-1.76016716
C	0.81776746	-0.43313470	0.02836156
C	-0.60074458	0.08329557	0.00877919
C	1.66190756	-0.19718530	1.23078307
C	1.55019060	-0.68152156	-1.24141456
C	-1.41320320	-0.50260862	1.17229931
C	-0.57651823	1.61800629	0.14716977
H	-1.59524094	2.01674177	0.12292476
H	-0.11367377	1.91537945	1.09000586
H	-0.01136654	2.06840361	-0.67282489

$Me_3CMe_2CMe_2C^{\bullet}$
E = -3800.0
H = -3613.1
G = -3646.9
N_{imag} = 0

C	-1.06679246	-1.19110991	-1.14248289
H	-0.76241450	-0.79895592	-2.11009096
H	-0.85234136	-2.26158565	-1.13221242
H	-2.14587101	-1.06678500	-1.05130203
H	-2.09454795	-0.89172298	1.27404773
H	-0.86413484	-2.15151692	1.34484630
H	-0.62687541	-0.64096112	2.21769826
H	2.92384461	-0.87299335	1.16795657
H	1.46951614	-0.63027585	2.14744323
H	1.86810132	-2.22987574	1.53023885
H	1.83572036	-2.35261169	-1.37370245
H	1.36661536	-0.82823291	-2.12132131
H	2.87046815	-0.94427349	-1.19534884
C	1.11868161	-0.88103499	0.02645692
C	-0.34804270	-0.51339645	0.03584543

C	1.86977557	-1.15355594	1.28048895
C	1.81996108	-1.25746268	-1.22963650
C	-1.01972550	-1.07149941	1.30028591
C	-0.51740165	1.06053377	-0.04296658
C	-1.98879292	1.47090144	-0.11770625
C	0.10776079	1.73381820	1.17965867
C	0.19593142	1.61926522	-1.27540888
H	-0.20710447	1.21173969	-2.20305861
H	0.06586941	2.70409216	-1.30739236
H	1.26424044	1.40622910	-1.23556355
H	-0.39364279	1.44892251	2.10509773
H	1.16586417	1.48517931	1.26175768
H	0.02213670	2.81884179	1.07821209
H	-2.46839672	1.09279072	-1.02118755
H	-2.55266994	1.12332013	0.74873913
H	-2.05573303	2.56221590	-0.13939234

$H_3CiPr_2CiPr_2C^{\bullet}$
E = -5667.7
H = -5387.0
G = -5427.5
N_{imag} = 0

C	2.22496572	-0.08838310	1.26211024
H	3.25027881	-0.05083261	0.88796433
C	2.20428616	-1.33291933	2.16054796
C	2.08607008	1.18055462	2.11787830
H	1.47218610	0.03828649	-2.09317650
C	2.95526973	1.34178352	-1.32654152
C	3.13250055	-1.14554920	-1.49353383
C	-1.08385410	0.29307686	-2.49260191
C	-0.22600998	-1.91437828	-1.73689579
H	-1.83770945	-0.82895402	-0.89816471
C	-2.20995426	0.12290238	1.51805957
C	-0.91134906	-1.97920901	1.34142815
H	-0.19906942	-0.12783118	2.09264782
C	-0.12947459	0.07800440	-0.03189319
C	1.37371610	-0.13507618	0.01348035
C	-0.83899935	-0.59148746	-1.26870169
C	-0.82006052	-0.45859977	1.26358227
C	2.16598153	0.02293128	-1.25861979
H	-1.23405657	-2.27428821	2.34375539
H	0.04221576	-2.45872305	1.14041041
H	-1.64563926	-2.36745122	0.63332296
H	1.28628423	-1.41742843	2.73922329
H	3.03877363	-1.27684877	2.86705773
H	2.32159724	-2.24160618	1.56768100
H	2.86521247	1.18467846	2.88690043
H	1.12281693	1.23660982	2.62578549
H	2.20223184	2.07685510	1.50819933
H	-2.18252340	1.19528574	1.71082925
H	-2.64902925	-0.36202610	2.39424689
H	-2.88526998	-0.05378378	0.67674223
H	3.72086897	1.38491258	-0.54899144
H	3.46125153	1.42161321	-2.29371199
H	2.30362636	2.20724083	-1.20638775
H	3.63292191	-1.03252024	-2.46022329
H	2.60796525	-2.10071745	-1.48280874
H	3.90660098	-1.17454143	-0.72313520
H	-0.15783102	0.65727193	-2.94100596
H	-1.60245774	-0.29856923	-3.25307954
H	-1.70728598	1.15747498	-2.26529018

H	-1.00390683	-2.54678096	-2.17453221
H	0.52728374	-1.75058783	-2.50700756
H	0.24455958	-2.46304438	-0.92715923
C	-0.36480306	1.60438769	-0.09450117
H	-1.42139492	1.83635190	-0.22520054
H	-0.02277268	2.07894250	0.82204876
H	0.17712131	2.03799124	-0.93114151

$Me_3CiPr_2CiPr_2C^{\bullet}$
E = -6769.7
H = -6433.1
G = -6477.7
N_{imag} = 0

C	2.29689525	0.23928998	1.20102584
H	3.26068305	0.37842841	0.70455589
C	2.48907344	-1.04304693	2.03844676
C	2.24150676	1.37979232	2.22191191
H	1.33855554	-0.42577375	-2.01438608
C	2.95598615	0.90390460	-1.79876850
C	2.91109082	-1.49819765	-1.13409652
C	-0.53098495	0.58942394	-2.65543156
C	-0.85895793	-1.63152169	-1.61329920
H	-1.92425296	0.10331890	-1.15116774
C	-2.33313076	-0.44560241	1.30191488
C	-0.36734897	-1.91775620	1.30527501
H	-0.42107544	0.05098069	2.08230643
C	-0.19139374	0.36653495	-0.00430182
C	1.32990438	0.15303844	0.02187532
C	-0.87817401	-0.12642408	-1.33142485
C	-0.80820035	-0.44769143	1.19606561
C	2.06632870	-0.22065483	-1.24589952
H	-0.20467376	-2.18025014	2.35212825
H	0.54872638	-2.11478871	0.76111718
H	-1.13436564	-2.59242644	0.92927186
H	1.67320571	-1.17014638	2.74759145
H	3.41575631	-0.94355045	2.61238175
H	2.56188951	-1.94032005	1.43215015
H	3.19480956	1.39422909	2.75895189
H	1.46639502	1.21060830	2.96966847
H	2.09627301	2.36038865	1.77752811
H	-2.77630652	0.54413547	1.32981411
H	-2.62014440	-0.96316741	2.22255264
H	-2.78307447	-0.99408957	0.47284959
H	3.72184399	1.20090470	-1.07777806
H	3.46889521	0.55723236	-2.70102646
H	2.37215088	1.78515928	-2.05432882
H	3.30949568	-1.75300213	-2.12062109
H	2.31656778	-2.34177688	-0.78055323
H	3.75829436	-1.37091518	-0.45855965
H	0.08565941	-0.03687369	-3.30181139
H	-1.45143834	0.79130565	-3.21046872
H	-0.00746266	1.52717656	-2.52529357
H	-1.26716852	-1.79776882	-2.61442743
H	0.14375932	-2.05358268	-1.59083154
H	-1.47528642	-2.19250126	-0.91906508
C	-0.59067080	1.94956757	0.18105039
C	-1.89373341	2.36408949	-0.52645878
C	-0.80551672	2.37358812	1.64336602
C	0.49975060	2.86978628	-0.38352641
H	0.50143589	2.88571365	-1.47047444
H	0.31479825	3.89430535	-0.05048460

H	1.49105769	2.57474673	-0.05893327
H	-1.67390294	1.90021928	2.09334905
H	0.04929909	2.18410318	2.27448835
H	-0.98534856	3.45137084	1.65702203
H	-1.83697384	2.28340313	-1.60748117
H	-2.75980613	1.80231736	-0.18325853
H	-2.07660805	3.41647370	-0.29175589

Table S7 Cartesian coordinates (in Å), ADF total energies (in kcal mol^{-1}), and number of imaginary frequencies of the stationary points of the radical addition reactions in this study, computed at ZORA-(U)OLYP/TZ2P.

$H_3C^•$
E = -406.5
H = -385.6
G = -399.1n
N_{imag} = 0

C	0.00000000	0.00000000	0.00000000
H	0.54151312	0.93792824	0.00000000
H	0.54151312	-0.93792824	0.00000000
H	-1.08302624	0.00000000	0.00000000

$Me_3C^•$
E = -1517.9
H = -1441.7
G = -1463.7
N_{imag} = 0

C	-0.00000000	-0.00000000	0.19160176
C	-0.74210328	1.28536060	0.02753075
C	-0.74210328	-1.28536060	0.02753075
C	1.48420657	-0.00000000	0.02753075
H	1.94641521	-0.88825457	0.47433073
H	1.94641521	0.88825457	0.47433073
H	1.78229526	-0.00000000	-1.04005946
H	-0.20395658	-2.12977231	0.47433073
H	-0.89114763	-1.54351297	-1.04005946
H	-1.74245864	-1.24151773	0.47433073
H	-1.74245864	1.24151773	0.47433073
H	-0.89114763	1.54351297	-1.04005946
H	-0.20395658	2.12977231	0.47433073

H_2CCH_2
E = -714.7
H = -680.8
G = -696.4n
N_{imag} = 0

C	0.00000000	0.00000000	-0.66611913
C	-0.00000000	0.00000000	0.66611913
H	-0.00000000	0.92584418	-1.23779373
H	0.00000000	-0.92584418	-1.23779373
H	-0.00000000	0.92584418	1.23779373
H	0.00000000	-0.92584418	1.23779373

Me_2CCMe_2
E = -2186.0
H = -2079.5
G = -2106.3
N_{imag} = 0

C	1.51623273	1.23500569	0.16252443
H	0.94458278	2.15270243	0.29573997
H	2.14502978	1.35709934	-0.73099827
H	2.20765066	1.15537500	1.01202481
H	1.97928304	-1.38939449	1.10413160
H	2.35341819	-1.18773364	-0.59941784
H	0.98246526	-2.18515896	-0.12118080
H	-2.35327724	-1.18802210	0.59952687
H	-0.98224068	-2.18526371	0.12114563
H	-1.97918602	-1.38949126	-1.10405631
H	-2.20763646	1.15529061	-1.01191707

H	-0.94478733	2.15265087	-0.29529159
H	-2.14529201	1.35674565	0.73114578
C	-0.67354545	-0.01630204	-0.06086217
C	0.67354741	-0.01624365	0.06101264
C	-1.52076315	-1.26754491	-0.11226803
C	-1.51634848	1.23486756	-0.16230356
C	1.52088340	-1.26740525	0.11233686

iPr_2CCiPr_2
E = -5092.0
H = -4838.4
G = -4878.5
N_{imag} = 0

C	1.60158426	1.23401485	0.00000000
H	2.61427354	0.82112093	0.00000000
C	1.55700339	2.09890464	1.27908607
C	1.55700339	2.09890464	-1.27908607
H	0.75615004	-2.14187760	0.00000000
C	2.32867117	-1.52410623	-1.26913180
C	2.32867117	-1.52410623	1.26913180
C	-1.55700339	-2.09890464	-1.27908607
C	-1.55700339	-2.09890464	1.27908607
H	-2.61427354	-0.82112093	0.00000000
C	-2.32867117	1.52410623	-1.26913180
C	-2.32867117	1.52410623	1.26913180
H	-0.75615004	2.14187760	0.00000000
C	-0.67940706	0.00874347	0.00000000
C	0.67940706	-0.00874347	0.00000000
C	-1.60158426	-1.23401485	0.00000000
C	-1.47212857	1.32448792	0.00000000
C	1.47212857	-1.32448792	0.00000000
H	-2.78192502	2.52211226	1.26018627
H	-1.72278584	1.44175956	2.17620050
H	-3.14711375	0.80167853	1.34674054
H	0.64998971	2.70120971	1.36621456
H	2.40679753	2.79220992	1.28582505
H	1.62861276	1.48000757	2.17776847
H	2.40679753	2.79220992	-1.28582505
H	0.64998971	2.70120971	-1.36621456
H	1.62861276	1.48000757	-2.17776847
H	-1.72278584	1.44175956	-2.17620050
H	-2.78192502	2.52211226	-1.26018627
H	-3.14711375	0.80167853	-1.34674054
H	3.14711375	-0.80167853	-1.34674054
H	2.78192502	-2.52211226	-1.26018627
H	1.72278584	-1.44175956	-2.17620050
H	2.78192502	-2.52211226	1.26018627
H	1.72278584	-1.44175956	2.17620050
H	3.14711375	-0.80167853	1.34674054
H	-0.64998971	-2.70120971	-1.36621456
H	-2.40679753	-2.79220992	-1.28582505
H	-1.62861276	-1.48000757	-2.17776847
H	-2.40679753	-2.79220992	1.28582505
H	-0.64998971	-2.70120971	1.36621456
H	-1.62861276	-1.48000757	2.17776847

TS_$H_3C^{\bullet}$+H_2CCH_2
E = -1113.6
H = -1057.9
G = -1078.7
N_{imag} = 1, ν = -363.9i cm^{-1}

C	-2.11181605	-0.68012232	-0.52522591
H	-3.14773586	-0.56220395	-0.82778846
H	-1.64778614	0.14603745	0.00178289
H	-1.77432007	-1.68008047	-0.27667307
C	-1.08303734	-0.42650005	-2.65582660
C	0.26457462	-0.53849019	-2.54646341
H	-1.55496469	0.55042596	-2.71123120
H	-1.68345040	-1.26805577	-2.98968559
H	0.89184167	0.32310296	-2.33087157
H	0.76282143	-1.50281895	-2.61045662

TS_Me$_3$C$^•$+H$_2$CCH$_2$
E = -2221.3
H = -2110.4
G = -2137.0
N_{imag} = 1, ν = -385.4i cm^{-1}

C	-2.07205317	-0.67313857	-0.59087879
C	-3.51950984	-0.51369941	-0.96727789
C	-1.48187504	0.45066062	0.21535005
C	-1.65760458	-2.05632559	-0.17108655
H	-0.57079200	-2.14623059	-0.08153513
H	-2.00982370	-2.82342884	-0.86891075
H	-2.08441201	-2.30359587	0.81629179
H	-0.39498874	0.36101825	0.30429702
H	-1.89112508	0.44597618	1.24039402
H	-1.71214802	1.43217978	-0.21250277
H	-3.72841028	0.46819120	-1.40533083
H	-4.16324929	-0.60503080	-0.07511320
H	-3.85150083	-1.28234799	-1.67337580
C	-1.07947413	-0.43032572	-2.61851340
C	0.28473173	-0.53160814	-2.59325659
H	-1.54032685	0.54945692	-2.71092438
H	-1.66963479	-1.26318688	-2.99128007
H	0.91717922	0.33225430	-2.40473600
H	0.78703691	-1.49115331	-2.68675404

TS_H$_3$C$^•$+Me$_2$CCMe$_2$
E = -2580.7
H = -2452.2
G = -2482.6
N_{imag} = 1, ν = -421.8i cm^{-1}

C	2.22896118	-0.06715240	-1.31045794
H	1.77837337	0.37503454	-2.19955331
H	2.35071734	-1.14358752	-1.49874409
H	3.23265745	0.35364335	-1.21065472
H	3.23083823	0.40286320	1.12492718
H	2.40902236	-1.13599382	1.37299403
H	1.78223529	0.33278718	2.12207833
H	-1.62535511	0.70614807	1.12549859
H	-0.23625913	0.25705912	2.12149320
H	-1.24555177	-0.98767364	1.38419820
H	-1.54834483	-0.86904593	-1.19020857
H	-0.21411320	-0.29633163	-2.19156081
H	-1.34069136	0.85261356	-1.45608045
C	0.04408096	-0.00367067	-0.03567907
C	1.41983605	0.15844859	-0.04191504
C	-0.78829652	-0.00060457	1.21817993
C	-0.78616405	-0.08467393	-1.28866205
C	2.24491338	-0.05995174	1.21647344
C	1.56080338	2.52077309	-0.05133913
H	2.63855449	2.65035366	-0.06645439

H	1.05891282	2.77928503	0.87559656
H	1.03461506	2.77010484	-0.96725932

TS_$Me_3C^{•}$+Me_2CCMe_2
E = -3679.8
H = -3496.0
G = -3530.9
N_{imag} = 1, ν = -434.6i cm^{-1}

C	2.26603940	-0.13178282	-1.24437323
H	1.92716893	0.28266638	-2.19430216
H	3.31308221	0.14879894	-1.11739758
H	3.19228534	0.38559745	1.23057767
H	-1.73648266	0.58939169	1.01929997
H	-0.14736410	-0.01704193	-2.26956955
H	-1.64402782	0.36139384	-1.40907042
C	0.03826102	0.04527428	-0.08728895
C	1.42874500	0.30007680	-0.04290567
C	-0.71364987	-0.22193094	-1.36215303
C	1.65806652	2.54794324	-0.16080740
C	3.13340070	2.84521270	-0.30848303
C	1.08002033	3.07907934	1.13125129
C	0.85303325	2.96286298	-1.37094085
H	1.22945556	2.52476115	-2.29970239
H	0.90637947	4.05738261	-1.49868447
H	-0.20473765	2.70789808	-1.27270133
H	1.64324387	2.75582832	2.01094848
H	0.03319820	2.79673566	1.26621762
H	1.11259368	4.18209084	1.12863497
H	3.55369796	2.45327920	-1.23836868
H	3.72955582	2.46889435	0.52781733
H	3.29027617	3.93810639	-0.33216971
H	2.24137162	-1.22582611	-1.34320524
H	2.24067989	-1.08205566	1.40149755
H	1.68894948	0.41311475	2.14657317
H	-0.34802431	0.25487211	2.06039853
H	-1.21148582	-1.06299818	1.27133741
H	-1.02625122	-1.27870226	-1.40962587
C	-0.83798337	-0.03479298	1.13204133
C	2.16902206	0.00541134	1.25783904

TS_$H_3C^{•}$+iPr_2CCiPr_2
E = -5472.1
H = -5196.0
G = -5238.3
N_{imag} = 1, ν = -422.4i cm^{-1}

C	2.23230291	-0.00781591	1.30027191
H	3.24668730	0.12315535	0.91442536
C	2.33552796	-1.29281176	2.15674978
C	2.03288423	1.22606293	2.20902400
H	1.53802098	-0.10797901	-2.10149768
C	2.98066983	1.29126609	-1.44329687
C	3.19614144	-1.22252715	-1.39124431
C	-1.00178552	0.37693741	-2.50248929
C	-0.38171807	-1.97930210	-1.90463804
H	-1.83977561	-0.77230918	-0.96877354
C	-2.19977698	0.07642600	1.49829939
C	-0.99328630	-2.10222172	1.39587392
H	-0.20500915	-0.25653067	2.10105861
C	-0.04172754	-0.16836938	-0.03470590
C	1.36358398	-0.10317494	0.02504014
C	-0.82041450	-0.60799800	-1.32807554

C	-0.82001616	-0.56883182	1.25913788
C	2.21119070	-0.03720249	-1.25154902
H	-1.33590328	-2.33913375	2.40925154
H	-0.06590337	-2.64911201	1.22451936
H	-1.74837678	-2.49128514	0.70665024
H	1.45803286	-1.46668640	2.78188153
H	3.19787421	-1.20746394	2.82861845
H	2.48941883	-2.18040571	1.53829519
H	2.83152809	1.24940158	2.95974476
H	1.08431942	1.22098019	2.75091740
H	2.09012499	2.15704388	1.64128591
H	-2.13692984	1.14961161	1.67447488
H	-2.65342348	-0.37234449	2.38895177
H	-2.89919643	-0.08875780	0.67288684
H	3.74748310	1.44973791	-0.67888130
H	3.49247875	1.28178886	-2.41239195
H	2.31447390	2.15615075	-1.43121325
H	3.67256991	-1.19259855	-2.37784443
H	2.69037857	-2.18615716	-1.29541041
H	3.99812600	-1.18996359	-0.64766692
H	-0.06300596	0.69604440	-2.96103236
H	-1.58587810	-0.11904835	-3.28645814
H	-1.54898034	1.27323035	-2.21337284
H	-1.19819001	-2.39269200	-2.50819909
H	0.48624034	-1.89752849	-2.56225779
H	-0.14339200	-2.70921746	-1.13189513
C	-0.49553410	2.13915687	-0.02182036
H	-1.55400243	2.22733283	-0.23925502
H	-0.20974284	2.40625514	0.98718233
H	0.16342421	2.50188726	-0.80026783

TS_$Me_3C^{\bullet}$+iPr_2CCiPr_2
E = -6547.3
H = -6215.4
G = -6261.9
N_{imag} = 1, ν = -366.1i cm^{-1}

C	2.34220567	0.10475245	1.23947941
H	3.31001629	0.29465204	0.76773039
C	2.58641457	-1.21098269	2.03195332
C	2.23421395	1.23227494	2.28514991
H	1.48209312	-0.22823801	-2.09503525
C	3.14091015	1.00679031	-1.67875156
C	2.99402973	-1.47142736	-1.31568075
C	-0.58120996	0.46196070	-2.69468972
C	-0.89577753	-1.77419324	-1.67528272
H	-1.89228948	-0.00706797	-1.11124415
C	-2.32901051	-0.39841604	1.36403383
C	-0.47414546	-2.00631927	1.49052931
H	-0.38268539	0.03759360	2.09673295
C	-0.09436272	0.14765533	-0.02230268
C	1.38166054	0.05568257	0.01594048
C	-0.85930306	-0.26133153	-1.34618724
C	-0.79409856	-0.50072441	1.24219460
C	2.18791468	-0.14690341	-1.27911747
H	-0.41219224	-2.19844004	2.56679555
H	0.45645618	-2.33590697	1.03887577
H	-1.26898211	-2.65241223	1.11151845
H	1.81311090	-1.39496337	2.77891419
H	3.53730800	-1.11757130	2.56966274
H	2.66024913	-2.09173618	1.39522688
H	3.13130466	1.20424562	2.91459732

H	1.38013445	1.11367700	2.95463381
H	2.19185287	2.22436970	1.83691194
H	-2.72528868	0.61068896	1.31134533
H	-2.62603206	-0.80712630	2.33698919
H	-2.84231977	-0.99872081	0.60773293
H	3.94977770	1.15978730	-0.95699318
H	3.61425785	0.76955975	-2.63848902
H	2.61681315	1.95472798	-1.79742664
H	3.38584236	-1.62667228	-2.32750527
H	2.37796853	-2.33830643	-1.06565160
H	3.85275086	-1.46133261	-0.63870610
H	0.07912182	-0.11562294	-3.34766787
H	-1.52490795	0.56431139	-3.24241465
H	-0.15195916	1.45436744	-2.59815497
H	-1.49930627	-1.91983728	-2.57901864
H	0.09595412	-2.18163371	-1.88314740
H	-1.34866287	-2.37925608	-0.89399118
C	-0.69210021	2.23460187	0.15933165
C	-2.00161820	2.49894106	-0.58447688
C	-0.87392554	2.61989787	1.62582671
C	0.41041476	3.09537256	-0.44318827
H	0.50688571	2.97787728	-1.52249590
H	0.17428901	4.15605814	-0.26672907
H	1.38520549	2.90994834	0.00365495
H	-1.65716906	2.06431229	2.14009745
H	0.03790969	2.53960240	2.20967634
H	-1.17352875	3.67876617	1.66575887
H	-1.93176353	2.35450198	-1.66099306
H	-2.84666679	1.91498259	-0.21840866
H	-2.27158193	3.55619091	-0.43286046

$H_3CH_2CH_2C^{\bullet}$
E = -1143.1
H = -1085.0
G = -1105.6n
N_{imag} = 0

C	-1.86106579	-0.63686593	-0.91696717
H	-2.95469509	-0.55872688	-0.92380894
H	-1.46842106	0.13071979	-0.24196552
H	-1.59136537	-1.61537593	-0.50618718
C	-1.29678791	-0.45998717	-2.34840649
C	0.18541725	-0.55066609	-2.43034148
H	-1.63205528	0.51501776	-2.72846855
H	-1.75328276	-1.22413617	-2.99234475
H	0.81028060	0.31862946	-2.24505277
H	0.68269965	-1.50935835	-2.54830059

$Me_3CH_2CH_2C^{\bullet}$
E = -2239.8
H = -2127.3
G = -2153.2n
N_{imag} = 0

C	-1.94097611	-0.64362321	-0.84823615
C	-3.46812341	-0.51233871	-0.99913372
C	-1.42499645	0.45114584	0.10378077
C	-1.60077658	-2.03358172	-0.27958212
H	-0.52223569	-2.15919720	-0.14525724
H	-1.95166430	-2.83211057	-0.94303093
H	-2.07699520	-2.18120268	0.69593373
H	-0.34241674	0.38146257	0.24607777
H	-1.89645251	0.36301912	1.08875613

H	-1.64911191	1.45221717	-0.28165778
H	-3.74734667	0.46725101	-1.40263735
H	-3.96816803	-0.62663592	-0.03098848
H	-3.87065162	-1.27731261	-1.67231408
C	-1.29799877	-0.46827946	-2.27886280
C	0.18055823	-0.55609270	-2.38290152
H	-1.63138431	0.50769664	-2.65722372
H	-1.75356828	-1.23097787	-2.92511688
H	0.80887192	0.32097693	-2.26255576
H	0.68073639	-1.50646216	-2.54144599

$H_3CMe_2CMe_2C^{\bullet}$
E = -2609.3
H = -2478.4
G = -2506.9
N_{imag} = 0

C	-1.33082331	-0.24082594	-1.31307839
H	-0.86039507	0.22845428	-2.18170679
H	-1.35714615	-1.32183866	-1.48358808
H	-2.36692203	0.11026833	-1.27345384
H	-2.44841282	-0.19393297	1.14607251
H	-1.41126573	-1.62837537	1.07213083
H	-0.99925521	-0.28518425	2.14496317
H	2.40299462	0.68150540	1.08111102
H	1.14026158	0.04659640	2.13675664
H	2.33522393	-1.03893802	1.42011233
H	2.48125602	-1.30817616	-1.00775679
H	0.98293453	-1.31769809	-1.94465015
H	1.92182483	0.16389085	-1.78991148
C	0.84117195	-0.37914767	0.01706148
C	-0.60606880	0.11025318	0.00393404
C	1.70240160	-0.16164960	1.22438663
C	1.57741178	-0.73215459	-1.23775764
C	-1.40798321	-0.53734157	1.16333632
C	-0.63945431	1.65648969	0.17491846
H	-1.67217343	2.02446985	0.16803149
H	-0.18306347	1.96890198	1.11839385
H	-0.10251721	2.15443285	-0.63930557

$Me_3CMe_2CMe_2C^{\bullet}$
E = -3693.6
H = -3508.0
G = -3542.0
N_{imag} = 0

C	-1.06699871	-1.20378405	-1.17807510
H	-0.72697365	-0.85004061	-2.15133519
H	-0.89936902	-2.28473276	-1.14742103
H	-2.14703897	-1.04716021	-1.13129396
H	-2.14212975	-0.94396923	1.22686441
H	-0.89590912	-2.17773307	1.34231575
H	-0.71686296	-0.66314281	2.21966673
H	2.91579878	-0.83657375	1.22920597
H	1.44145089	-0.75766320	2.19719443
H	1.97010429	-2.28699116	1.50215895
H	1.96378723	-2.40909308	-1.30682828
H	1.43220418	-0.94548563	-2.12944877
H	2.91070014	-0.94086470	-1.16457840
C	1.12697914	-0.92975065	0.03791266
C	-0.35206316	-0.52633078	0.02346376
C	1.88192224	-1.20232308	1.30486385
C	1.87650545	-1.31137826	-1.20386051

C	-1.06206169	-1.09781159	1.28166313
C	-0.52111852	1.08817345	-0.04587187
C	-2.00736875	1.51518469	-0.06375183
C	0.14652357	1.77606035	1.16596722
C	0.14550962	1.66938164	-1.31288722
H	-0.30276938	1.29428249	-2.23631646
H	0.02731163	2.75820595	-1.32222933
H	1.21710806	1.45969802	-1.34232835
H	-0.30170843	1.48252941	2.11849268
H	1.21796157	1.56880600	1.21305513
H	0.02927140	2.86172971	1.08126402
H	-2.54505728	1.12903373	-0.93323019
H	-2.54497075	1.20375185	0.83526016
H	-2.07073814	2.60799125	-0.10989246

$H_3CiPr_2CiPr_2C^{\bullet}$

E = -5498.0

H = -5219.6

G = -5261.2

N_{imag} = 0

C	2.25057411	-0.05832969	1.31194918
H	3.27342708	0.02651092	0.93293316
C	2.29794327	-1.34223378	2.17855885
C	2.10281426	1.18190272	2.22986662
H	1.54535810	-0.02493111	-2.09136141
C	3.02361887	1.32293100	-1.39608575
C	3.20222416	-1.18907638	-1.44866481
C	-1.07770985	0.32164959	-2.56707876
C	-0.32457734	-1.94162445	-1.82338411
H	-1.87079382	-0.77402718	-0.96898725
C	-2.27080492	0.08581534	1.53945255
C	-0.97763951	-2.03283095	1.35571644
H	-0.25493306	-0.17745670	2.09606454
C	-0.14273384	0.07239293	-0.04464569
C	1.39747942	-0.10631693	0.03408260
C	-0.85658189	-0.57745446	-1.33029783
C	-0.87101202	-0.49956600	1.25565252
C	2.22887734	-0.00176095	-1.24488555
H	-1.29045583	-2.30808623	2.36907095
H	-0.03559100	-2.54257750	1.15894455
H	-1.73021883	-2.43853057	0.67349298
H	1.40592082	-1.48231390	2.79143005
H	3.15291665	-1.28213790	2.86268445
H	2.43163676	-2.23674520	1.56521158
H	2.90620689	1.17381621	2.97605261
H	1.15867239	1.20645754	2.77976839
H	2.18897948	2.11458876	1.66739121
H	-2.25557399	1.16104382	1.72716369
H	-2.68073331	-0.38801739	2.43796673
H	-2.98470933	-0.10374117	0.73071480
H	3.80273716	1.43039148	-0.63517970
H	3.52271588	1.34513820	-2.37197428
H	2.37711481	2.20137950	-1.33675783
H	3.67523933	-1.12064133	-2.43524237
H	2.68900866	-2.15130845	-1.39048966
H	4.00739814	-1.19508085	-0.70749100
H	-0.14919953	0.63587776	-3.05227400
H	-1.64729223	-0.24466626	-3.31291395
H	-1.65568279	1.22064714	-2.34547511
H	-1.13442293	-2.48634190	-2.32232525
H	0.47460289	-1.83109466	-2.55942679

H	0.05125337	-2.57792518	-1.02511086
C	-0.40437507	1.61292993	-0.07620590
H	-1.46027941	1.84595005	-0.23017149
H	-0.09942897	2.08115995	0.85972003
H	0.15173642	2.09176854	-0.88158810

$Me_3CiPr_2CiPr_2C^•$
E = -6555.2
H = -6221.9
G = -6268.1
N_{imag} = 0

C	2.32453170	0.22157339	1.26812124
H	3.19322018	-0.35940491	0.94554555
C	1.98588621	-0.41237163	2.63944818
C	2.94768536	1.61923292	1.56059649
H	1.45795397	-0.44820524	-2.01957032
C	3.21363861	0.67900214	-1.71618840
C	2.86057315	-1.73051117	-1.11226403
C	-0.55441551	0.74747536	-2.72725990
C	-0.85984325	-1.58527768	-1.93627152
H	-1.90933000	0.09434927	-1.27075180
C	-2.37479705	-0.81621924	1.18334056
C	-0.32602606	-2.20145812	1.09041573
H	-0.50243058	-0.31433846	2.00712869
C	-0.18948561	0.24824557	-0.04194780
C	1.37751649	0.08243997	0.03893762
C	-0.85625767	-0.10849950	-1.46765964
C	-0.83688734	-0.73899018	1.06460432
C	2.16669978	-0.34528935	-1.20817831
H	-0.39526924	-2.58114729	2.11570926
H	0.70839558	-2.30428777	0.77299586
H	-0.92743768	-2.87174551	0.47554060
H	1.20214806	0.11239653	3.18937235
H	2.88560708	-0.36694620	3.26377233
H	1.70582423	-1.46150952	2.56290567
H	3.83621633	1.47524828	2.18656580
H	2.27756493	2.28085614	2.11264642
H	3.27045843	2.13860672	0.65914252
H	-2.84607351	0.12535204	1.45904359
H	-2.62191368	-1.52937005	1.97854422
H	-2.86058610	-1.18322233	0.27544270
H	4.07364668	0.76399090	-1.04620408
H	3.59859281	0.35350580	-2.68975389
H	2.78547075	1.67482613	-1.84721826
H	3.27959704	-1.99372571	-2.09031256
H	2.16867493	-2.52628693	-0.82984180
H	3.69134856	-1.74388198	-0.39987814
H	0.38694301	0.49628385	-3.22042590
H	-1.34555037	0.54973305	-3.45987451
H	-0.55266251	1.81965211	-2.55863483
H	-1.30670064	-1.62833353	-2.93558419
H	0.12785513	-2.03907296	-2.01383031
H	-1.46810912	-2.22686365	-1.30312822
C	-0.64729570	1.82084845	0.32254553
C	-2.05375124	2.23048398	-0.20541023
C	-0.69489017	2.10199001	1.84571208
C	0.33279326	2.86768811	-0.26523522
H	0.51722877	2.74604396	-1.32969203
H	-0.09043448	3.86871381	-0.12520229
H	1.29534509	2.85791414	0.23342918
H	-1.52577463	1.60415337	2.34823284

H	0.22173034	1.82853536	2.36101526
H	-0.83529552	3.17720911	2.00026285
H	-2.09991384	2.32973966	-1.28951585
H	-2.85668914	1.56258628	0.10075313
H	-2.29353829	3.21758091	0.20496896
